# Comprehensive genetic testing of Chinese SNHL patients and variants interpretation using ACMG guidelines and ethnically matched normal controls

**DOI:** 10.1038/s41431-019-0510-6

**Published:** 2019-09-20

**Authors:** Yongyi Yuan, Qi Li, Yu Su, Qiongfen Lin, Xue Gao, Hankui Liu, Shasha Huang, Dongyang Kang, N. Wendell Todd, Douglas Mattox, Jianguo Zhang, Xi Lin, Pu Dai

**Affiliations:** 1 Departments of Otolaryngology Head&Neck Surgery, Chinese PLA General hospital, 28#Fuxing Road, 100853 Beijing, China; 2 Departments of Otolaryngology Head&Neck Surgery, Emory University School of Medicine, 615 Michael Street, Atlanta, GA 30322 USA; 3 Departments of Otolaryngology Head&Neck Surgery, Southern Medical University Nanfang Hospital, 1838# North Guangzhou Avenue, 510515 Guangzhou, China; 4 BGI-Shenzhen, 518083 Shenzhen, China; 5 China National GeneBank, BGI-Shenzhen, 518120 Shenzhen, China; 6 Department of Otolaryngology, PLA Rocket Force Characteristic Medical Center, 100088 Beijing, China

**Keywords:** Disease genetics, Medical genomics

## Abstract

Hereditary hearing loss is a monogenic disease with high genetic heterogeneity. Variants in more than 100 deafness genes underlie the basis of its pathogenesis. The aim of this study was to assess the ratio of SNVs in known deafness genes contributing to the etiology of both sporadic and familial sensorineural hearing loss patients from China. DNA samples from 1127 individuals, including normal hearing controls (*n* = 616), sporadic SNHL patients (*n* = 433), and deaf individuals (*n* = 78) from 30 hearing loss pedigrees were collected. The NGS tests included analysis of sequence alterations in 129 genes. The variants were interpreted according to the ACMG/AMP guidelines for genetic hearing loss combined with NGS data from 616 ethnically matched normal hearing adult controls. We identified a positive molecular diagnosis in 226 patients with sporadic SNHL (52.19%) and in patients from 17 deafness pedigrees (56.67%). Ethnically matched MAF filtering reduced the variants of unknown significance by 8.7%, from 6216 to 5675. Some complexities that may restrict causative variant identification are discussed. This report highlight the clinical utility of NGS panels identifying disease-causing variants for the diagnosis of hearing loss and underlines the importance of a broad data of control and ACMG/AMP standards for accurate clinical delineation of VUS variants.

## Introduction

Hearing impairment is one of the most common human disabilities. According to the World Health Organization, 5% of the world’s population (~360 million people) suffer from disabling hearing. More people are affected by severe hearing loss than by epilepsy, multiple sclerosis, spinal injury, stroke, Huntington’s disease, and Parkinson’s disease combined [1]. Sensorineural hearing loss (SNHL) accounts for ~90% of all human hearing loss cases. Among these patients, genetic factors are estimated to be responsible for >60% of the cases [2, 3], and most of these cases are caused by a single nucleotide variants (SNVs), a small fraction by a small insertion–deletion (indel) variant or copy number variants (CNVs) [4, 5]. Depending on the gene involved, the hearing loss can either be syndromic or nonsyndromic. A genetic diagnosis is valuable for providing essential prognostic information needed for deciding optimal treatment/rehabilitation options, and is needed for genetic consulting to predict the risk of recurrence [6].

Molecular epidemiological studies have found several common deafness genes, such as *GJB2*, *SLC26A4*, and mitochondrial *12S rRNA*, which appear to account for 30–50% of congenital hearing loss cases [7–12]. However, genetic variants responsible for a large number of cases of hereditary hearing loss remain unknown, especially in patients with sporadic hearing loss. Next-generation sequencing [2] has greatly increased efficiency in screening known deafness genes for diagnostic purposes and in identifying new deafness genes [5, 13–15]. However, the accuracy and clinical utility of the NGS approach has not been systematically evaluated in a large number of clinically diagnosed cases of sporadic SNHL and deafness pedigrees of Chinese ethnicity. This translational study employed NGS to screen 119 nuclear deafness genes and 10 mitochondria genes in patients with sporadic SNHL, individuals from deafness pedigrees, and ethnically matched normal hearing controls. The data were used to systematically assess the diagnostic rate of the deafness genes panel and the role of ethnically matched controls in interpreting variants to eliminate false positives.

## Materials and methods

### Collection of patient and control samples

This study was performed according to the protocol approved by the Ethics Committee of the Chinese People’s Liberation Army General Hospital. We collected DNA samples from 1127 individuals, including normal hearing controls (*n* = 616), sporadic patients clinically diagnosed SNHL (*n* = 433), and individuals from 30 deaf pedigrees including familial cases (*n* = 78). Ninety-nine family members with normal hearing from the 30 deaf pedigrees were enrolled for segregation analysis.

The sporadic cases were recruited from outpatients who visited the hospital during 2011–2013; only unique probands were included (each nuclear and/or extended family was represented only by the proband). The patients comprised those who claimed hearing loss and were verified by either subjective and/or objective hearing tests, those who failed the newborn hearing screening and were further diagnosed as deafness by objective hearing tests, and those who failed the newborn deafness gene screening carrying at least one variant of the nine including *GJB2* c.35delG, c.176del16, c.235delC, c.299delAT (NM_004004.5)*, GJB3* c.538C>T (NM_001005752.1)*, SLC26A4* c.919-2A>G (NG_008489.1), c.2168A>G (NM_000441.1)*, mtDNA 12S rRNA*, and were further referred to the otology clinic and diagnosed as hearing impairment by objective hearing tests. Chinese individuals with normal hearing (controls) were verified by pure-tone audiometry. Only those with a PTA (pure-tone average) threshold of 0–20 dB were enrolled. The average age of this population was 31.18 ± 10.18 years; no control individuals claimed hearing problems or family history, and all passed physical examinations. All participants were informed about the scope and requirements of the study, and all signed the consent forms approved by the Ethics Committees of the participating institutions.

The clinical history was obtained by investigators, with special emphasis on the onset age of hearing loss; family history of deafness; pregnancy and labor history; general health condition; potential environmental causes of hearing loss such as infections and trauma; and information on exposure to known or possible ototoxic drugs. Cases involving trauma and otitis media were excluded from this study. Conventional clinical examinations included pure-tone audiometry, acoustic immittance, and auditory brainstem responses (ABR). Distortion product otoacoustic emissions, ABR, and auditory steady state response tests were carried out in babies who failed the hearing screening and in children who were uncooperative during the subjective audiometry examination. The level of hearing loss was described in terms of PTA (calculated as the average of the threshold measured at 0.5, 1.0, 2.0, and 4.0 kHz) as follows: normal hearing, <20 dB; mild hearing impairment, 21–40 dB; moderate hearing impairment, 41–70 dB; severe hearing impairment, 71–90 dB; and profound hearing impairment, >91 dB. High-resolution computed tomography (CT) of the temporal bone was performed in the sporadic and pedigree SNHL cases to exclude middle ear pathological changes and to diagnose inner ear malformation. Magnetic resonance imaging was performed when the CT scan failed to reveal membranous labyrinth problems in a patient. Syndromic hearing loss was diagnosed based on a range of evidence, including self-reported symptoms, family history, and physical examination, with special attention to the external ears and neck, skin, hair, eyes, and digits. Data Management System for Clinical Otology software (Computer Software Copyright: 2008SR06229) was used to store and track the information.

### DNA isolation and previous genetic test

The DNA was isolated from blood extracted from a peripheral vein by a registered nurse in the genetic testing center of the Chinese PLA General Hospital. Genomic DNA (gDNA) was extracted using gDNA blood extraction kits (Qiagen, Valencia, CA, USA) within 1 week of sample collection. The quality of the gDNA was examined by assessing the optical density ratio (260/280 ratio) and by gel electrophoresis imaging for the presence of a high-molecular-weight gDNA band. All of the 433 sporadic cases were sequenced *12S rRNA*, the coding areas of *GJB2 and SLC26A4* previously.

### Library preparation and sequencing

The DA3 panel assay (Otogenetics Corporation, Atlanta, GA) includes 119 nuclear deafness genes and 10 mitochondria genes (Table S1). Library preparation and sequencing were described in our previous report [16].

### Bioinformatics

After sequencing the target region, quality control was performed to ensure data accuracy. Low-quality data were filtered out to obtain clean sequencing data. Burrows–Wheeler alignment was used to align the clean sequence to the human reference genome hg19 nuclear genes or GRCh37 for mitochondria genes. GATK was used to detect SNP and Indel variants. VEP and dbNSFP databases were used to obtain the variants information, including minor allele frequency (MAF), variant consequence, altered protein function, gene information, and related disease information.

### Statistical analysis

After raw variants were annotated, we performed further filtering to identify candidate variants. Variants meeting all the following requirements were included: (1) MAF of KG and Exome Aggregation Consortium (ExAC) database < 0.01; (2) 0.005 < MAF < 0.05; (3) variants affecting protein function, including loss-of-function and missense variants. After that, we obtained 142 variants from 433 hearing loss cases and 616 normal controls. We performed a case-control association study using Fisher’s exact test and adjusted *p*-values using Bonferroni multiple testing correction. Variants with *p*-values less than 3.521^E−04^ (0.05/142) were considered to be significantly associated with disease.

**Fig. 1:**
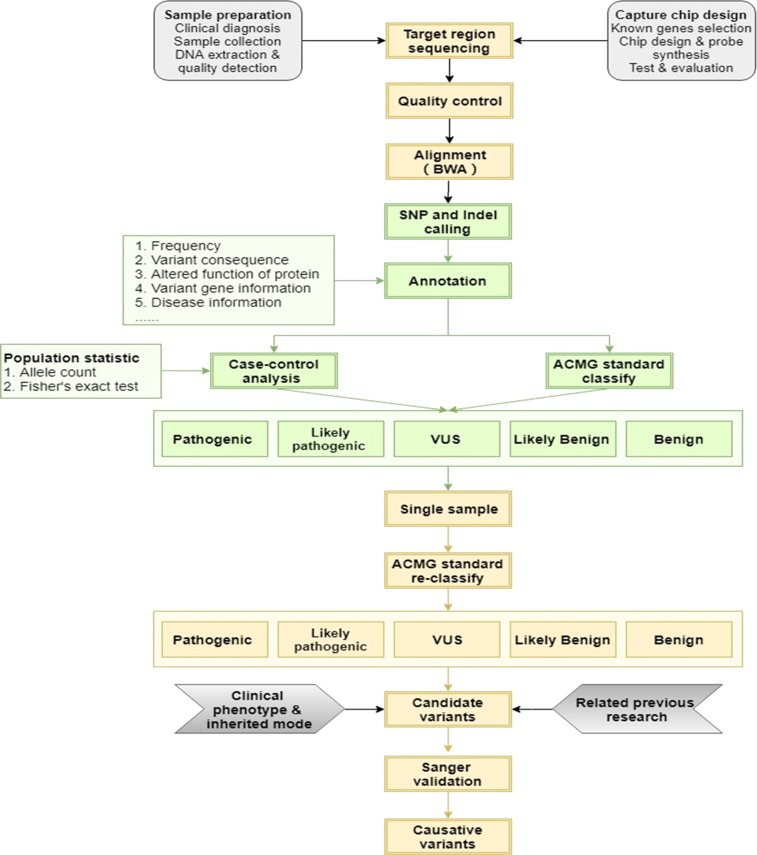
The pipeline of bioinfomatic analysis

The variants interpreted by the ACMG/AMP guidelines for genetic hearing loss were revalued using allele frequencies from the ethnically matched controls. The variants were categorized as BA1 if the MAF thresholds of autosomal-recessive variants were >0.005 and autosomal-dominant variants were >0.001 in the controls [17, 18]. The above thresholds >0.005 of autosomal-recessive variants excluded specific variants in *GJB2* and *SLC26A4*. By combining the clinical phenotype, inherited model, and related previous studies, we obtained a list of candidate variants. Sanger analysis was performed to verify the variant identified by NGS. The pipeline of bioinformatic analysis was shown in Fig. 1.

The FASTQ data and variant call format were permanently reserved on (CNSA). The pathogenic and likely pathogenic variants were submitted to ClinVar (ClinVarAccessions: SCV000902290~SCV000902403).

## Results

The data mapped to the targeted region have a mean depth of 149.027 fold, and the coverage of 1X was 99.8%. The capture rate is nearly 100%.

The phenotypic information of all patients and controls were shown in Table 1.

**Table 1:** General information and phenotypic characteristic of sporadic deafness patients and normal hearing controls

	Sporadic patients		Controls
Characteristic	number	percentage of the total	number
Sex			
Male	232	53.58%	345
Female	201	47.42%	271
Age when DNA samples collected			
Average	10.55		31.18
Age ≤ 2	73	16.86%	
Age 3–17	259	59.82%	
Age ≥ 18	101	23.32%	616
Ethnicity			
Han	415	95.84%	616
Hui	3	0.92%	
Man	9	2.08%	
Dai	1	0.23%	
Mongolia	3	0.69%	
Zhuang	1	0.23%	
Xibo	1	0.23%	
Family history			
No Deafness family history	407	94.00%	
Report deafness family history	14	3.23%	
Ambiguous	12	2.77%	
Report use of ototoxic drugs	7	1.62%	
Onset of hearing loss			
Congenital	317	73.21%	
Childhood	101	23.33%	
Adult	3	0.69%	
Ambiguous	12	2.77%	
Severity			
Normal			616
Mild-moderate	13	3.00%	
Severe-profound	408	94.23%	
Without audiogram	12	2.77%	
Laterality			
Bilateral	432	99.77%	
Unilateral	1	0.23%	
Syndromic hearing loss	3	0.69%	
Nonsyndromic hearing loss	430	99.31%	
Temporal bone computed tomography			
Bilateral enlarged vestibular aqueduct	89	20.55%	
Other inner ear malformation	11	2.54%	
Normal	333	76.91%	
Physical examination			
Microtia	2	0.46%	
Goiter	1	0.23%	
Heterochromia iridis	2	0.46%	
Cutaneous pigmentation	2	0.46%	
Ocular hypertelorism	1	0.23%	
Cochlear implantation	121	27.94%	

In the 119 nuclear deafness genes, variants in exon or splicing regions (totaling 6185) and intron regions (totaling 16521) were called after the preliminary bioinformatic analysis. We identified 73 (73/6185, 1.18%) pathogenic and 49 (49/6185, 0.79%) likely pathogenic variants (Table S2). In all, 1804 (1804/6185, 29.17%) variants were classified as benign, 919 (919/6185, 14.86%) as likely benign, and 3340 (3340/6185, 54.00%) variants were classified as uncertain significance. As for the definitely deafness-related mitochondrial variants, four homoplasmic m.1555A>G and one homoplasmic m.1494C>T carriers were found in the case group, and one homoplasmic m.1555A>G carrier was identified in the control group. Of the five patients carrying m.1555A>G or m.1494C>T, history of aminoglycoside antibiotics usage due to infection was clear.

### Variants classification and evaluation

We chose 1000 Genomes, Exome Sequencing Project [19], ExAC, HapMap, and Wellderly plus five in-house databases for MAF filtering. A total of 8.75% (541/6184) of exon variants were filtered out by the NGS data from the ethnically matched controls. After Bonferroni correction, 6 variants (*GJB2* c.235delC, c.299_300delAT, *SLC26A4* c.919-2A>G, c.2168A>G, c.1174A>T, *USH2A* c.6998T>C) achieved a conservative significance threshold. Among the 541 variants, the 1000 Genomes, ESP, ExAC databases reported no MAF thresholds for autosomal-recessive variants >0.005 (excluding specific variants in *GJB2* and *SLC26A4*) and autosomal-dominant variants >0.001; however, MAF > 0.005 was found in our control group in the recessive inheritance pattern and >0.001 in the dominant inheritance pattern and accordingly we categorized them to BA1. This result illustrated the power of this step and stressed the need for deeper and richer databases of multiple ethnicities to identify low-frequency variants specific to ancestry groups that are often unique to specific populations [20].

The allele frequency for *GJB2* c.109G>A (p.Val37Ile) was 3.8% (33/866, 1 homozygote and 31 heterozygotes) and 3% (37/1232, 1 homozygote and 35 heterozygotes) in the case and control groups, respectively; the difference between the two groups was not statistically significant. The homozygous p.V37I variant in *GJB2* is prevalent in East and Southeast Asians and may lead to a broad spectrum of hearing phenotypes from mild-to-moderate hearing loss with reduced penetrance to profound hearing loss. The homozygous p.V37I knockin mice developed progressive, mild-to-moderate hearing loss over the first 4–9 months which modeled the hearing phenotype of the human patients, and confocal immunostaining and electron microscopic scanning revealed minor loss of the outer hair cells [21]. Thus, we regard it as likely pathogenic variants. The fact that the majority of the case cohort showed severe to profound hearing loss may be the explanation for the lack of statistical significance. This result suggests that full-scale evaluations of the pathogenicity of a variant must include molecular epidemiology, functional experiments, and information from published studies.

### Positive diagnostic rate for SNVs

**Fig. 2:**
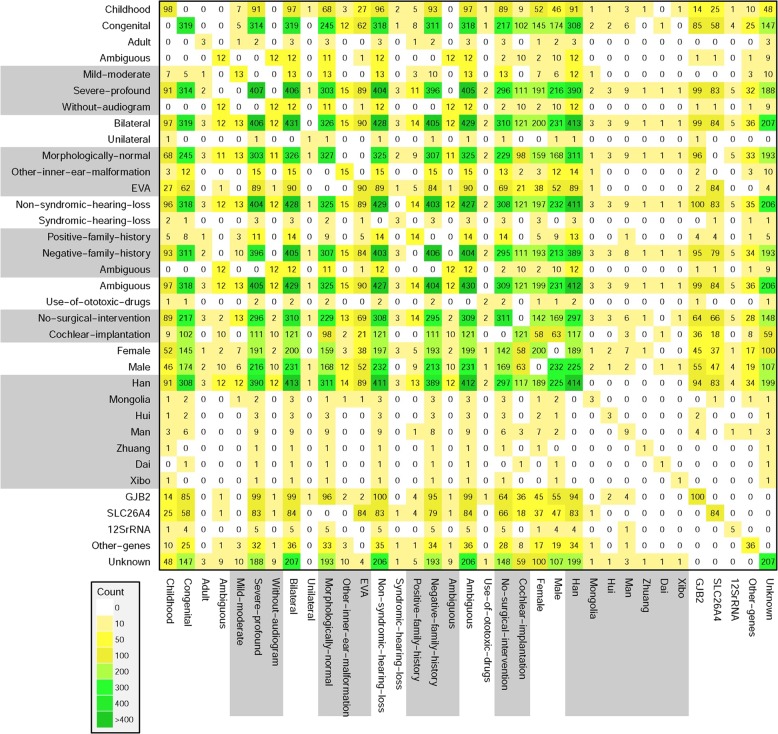
The interaction graph of basic and diagnostic information for each sample. Positive diagnosis is influenced by ethnic, clinical, and phenotypic characteristics in sporadic hearing loss population. *N* for each combination of two reported characteristics for all combinations. Color/shading reflects the number of patients with the paired criteria, up to the maximum of *n* = 433

In sporadic SNHL cases, variants in 24 genes were verified for genetic etiology in 52.19% (226/433) by Sanger sequencing and cosegregation analysis in their families (Table 2). The basic and diagnostic information for each sample were shown in Fig. 2. Considering the ratio of hereditary factors in hearing loss (60%), the positive diagnostic rate of the gene panel containing 129 genes in hereditary hearing loss might reach 86.98% (52.19%/60%). Detailed genotypes are shown in Table S3. Possible causative variants were found in 7.39% (32/433) of the cases; however, DNA samples and exact phenotypes of the family members were not available, and the genetic diagnosis could not be confirmed. Although 119 or more genes were included in clinical genetic testing, a distinct hotspot gene spectrum focused on several genes including *GJB2*, *SLC26A4*, *12S rRNA*, and *MYO15A*. The allele and carrier frequencies for variants in common deafness gene are shown in Table S4.

**Table 2:** SNVs Diagnoses Rate and Inheritance Patterns in Sporadic Patients with Genetic Hearing Loss

Gene	Total diagnoses	Autosomal dominant	Autosomal recessive	Mitochondrial	X-Linked	
	Diagnoses	%	Diagnoses	Diagnoses	Diagnoses	Diagnoses
*GJB2*	100	23.09%		100		
*SLC26A4*	84	19.39%		84		
*12SrRNA*	5	1.15%			5	
*MYO15A*	4	0.92%		4		
*POU3F4*	3	0.69%				3
*USH2A*	3	0.69%		3		
*MYO1F*	3	0.69%		3		
*MYO7A*	3	0.69%		3		
*TMC1*	3	0.69%		3		
*TRIOBP*	2	0.69%		2		
*CDH23*	2	0.46%		2		
*KCNQ4*	2	0.46%	2			
*ADGRV1*	1	0.23%		1		
*PAX3*	1	0.23%	1			
*MITF*	1	0.23%	1			
*CLDN14*	1	0.23%		1		
*OTOA*	1	0.23%		1		
*OTOF*	1	0.23%		1		
*PDZD7*	1	0.23%		1		
*TECTA*	1	0.23%		1		
*TRPN*	1	0.23%		1		
*TMPRSS3*	1	0.23%		1		
*KCNQ1*	1	0.23%		1		
*MYO6*	1	0.23%	1			
Total	226	52.19%	5	213	5	3

In deafness pedigrees, variants affecting protein function in 12 genes were verified with the positive diagnostic rate 56.67% (17/30) (Table 3). Detailed information on these deafness pedigrees is provided in our previous studies [22–29].

**Table 3:** Genotypes of deafness pedigrees

Family	Inheritance patterns	Genes	Gene accession numbers	Nucleotide changes	Amino acid changes	No. of patients^a^	No. of Normal hearing family members^a^
1	AD	*EYA4*	NM_004100.4 NP_004091.3	c.1364delG	p.(Gly455^a^)	3	1
2	AD	*GSDME*	NG_011593.1 NM_004403.2 NP_004394.1	c. 991-1G>C	Splicing site	3	3
3	AD	*TMC1*	NM_138691.2 NP_619636.2	c.1714G>A	p.(Asp572Asn)	3	3
4	AD	*ACTG1*	NM_001614.4 NP_001605.1	c.638A>G	p.(Lys213Arg)	5	2
5	AD	*EYA4*	NM_004100.4 NP_004091.3	c.544insA	p.(Phe221^a^)	7	8
6	AD	*KCNQ4*	NM_004700.3 NP_004691.2	c.887G>A	p.(Gly296Ala)	4	2
7	AD	*POU4F3*	NM_002700.2 NP_002691.1	c.602T>C	p.(Leu201Pro)	2	1
8	AR	*MYO7A*	NM_000260.3 NP_000251.3	c.[73G>A]; [462C>A]	p.(Gly25Arg); (Cys154^a^)	3	4
9	AR	*TMPRSS3*	NM_024022.2 NP_076927.1	c.[916G>A]; [36delC] c.[916G>A]; [316C>T]	p.(Ala306Thr); (Phe13Serfs∗12) p.(Ala306Thr); (Arg106Cys)	2	2
10	AR	*TECTA*	NM_005422.2 NP_005413.2	c.[257C>G]; [260_262delTTC]	p.(Ser86Cys); (Pro88del)	4	8
11	AR	*CDH23*	NM_022124.5 NP_071407.4	c.[6220delC]; [1117G>A]	p.(Leu2074^a^); (Val373Met)	2	2
12	AR	*SLC26A4*	NG_008489.1 NM_000441.1 NP_000432.1	c.[919-2A>G]; [1226G>A]	Splicing site; p.(Arg409His)	2	2
13	AR	*SLC26A4*	NG_008489.1 NM_000441.1 NP_000432.1	c.[1614-6T>G]; [1240_1243delinsGAGA>AAAG]	Splicing site; p.(Glu414_Ser415delinsLysGly)	2	2
14	AR	*SLC26A4*	NM_000441.1 NP_000432.1	c.[1226G>A]; [1340delA]	p.(Arg409His); (Lys447Serfs*8)	2	2
15	AR	*MYO7A*	NM_000260.3 NP_000251.3	c.[1991C>T]; [3799G>A]	p.(Thr664Ile); (Gly1267Arg)	2	0
16	X-linked	*POU3F4*	NM_000307.4 NP_000298.3	c.973delT	p.(Trp325Glyfs*12)	2	4
17	X-linked	*POU3F4*	NM_000307.4 NP_000298.3	c.927_929delCTC	p.(Ser310del)	4	11

^a^Number of patients or normal hearing family members who were tested by DA1 or/and DA3 panel. The DA1 or DA3 (Otogenetics Corporation, Atlanta, GA) panel includes targeted capture of 43 deafness genes and 119 deafness genes, respectively. For the list of 119 genes please see Table S1. Family 11–16 were tested by DA1 primitively and when DA3 came out, they were tested again as positive samples. The results either by DA1 or by DA3 are consistent

## Discussion

Identification of the precise genetic cause of hearing loss can provide helpful information for treatment like cochlear implantation [30], and patient management such as hearing and speech rehabilitation, prediction of prognosis, genetic counseling, and precise genetic therapies [19]. Previously, no comprehensive diagnostic testing had been completed in a Chinese deafness cohort, and no related gene variant data had been obtained in normal hearing controls with PTA-verified tests. To determine the aggregate genetic contribution to SNHL, we performed clinical genetic testing on 119 nuclear deafness genes and 10 mitochondria genes in 433 sequentially recruited patients with sporadic hearing loss, 616 controls, and 30 deafness pedigrees. No sporadic patients were excluded based on phenotype, inheritance, or previous testing.

The comprehensive testing resulted in the identification of gene sequence alterations associated with the underlying genetic cause for hearing loss in 226 patients (52.19%), higher than the 41% identified among cases with multiethnic background [15] and ~40% among Japanese patients [31]. Although Christina et al. [15] reported a diagnostic rate of 1% in patients with unilateral hearing loss (69 cases), no diagnostic positive result was found in the patient with unilateral hearing loss in our study. The negative result might be explained by the small sample of cases with unilateral hearing loss. In the 226 patients with positive diagnoses, the percentages of recessive, dominant, mitochondrial, and X-linked inheritance patterns were 94.24% (213/226), 2.21% (5/226), 2.21% (5/226), and 1.33% (3/226), respectively. Causative dominant variants were relatively less frequently identified in sporadic hearing loss. In our study, 7.39% (32/433) of our cases with likely causative variants could not be genetically diagnosed due to the lack of DNA samples or exact phenotypes from family members. In addition, some of our assumed de novo variants lacked confirmation of paternity and maternity.

### Genetic spectrum in the Chinese population

**Fig. 3:**
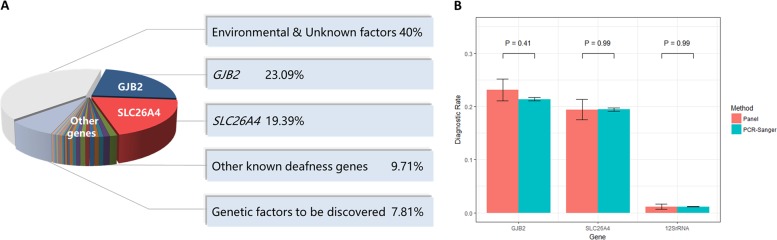
Etiology classification and the representation analysis of the studied sporadic patient cohort. **a** Pathogenesis of 433 Chinese sporadic hearing loss patients. **b** The representation comparison of the studied 433 cases with 16,456 patients from our Clinic. Salmon pink indicated the positive diagnostic ratio of deafness panel (including 119 genes and mitochrodrial genome) in 433 cases. Light green indicated the positive diagnostic ratio of Sanger sequencing for common deafness genes (*GJB2*, *SLC26A4,* and mit*12S rRNA*) in 16,456 cases. The first three pairs of columns showed there are no significant differences of the positive testing ratio on *GJB2, SLC26A4*, mit*12S rRNA* between the two patient cohorts, which indicated that the cases enrolled in this study could represent a larger deafness population in China. The largest ethnic group in both cohorts was Han Chinese, comprising up to 95% of the total sample

Despite the high genetic heterogeneity of hearing loss, most disease-causing variants are rarely recurrent [32] except those in *GJB2* and *SLC26A4*. Our study showed that disease-causing variants in *GJB2 and SLC26A4* were found in exceptionally high numbers, followed by variants in *USH2A*, *MYO15A*, *MYO1F*, and *MYO7A*. Variants affecting protein function in *GJB2* and *SLC26A4* were the most common cause of autosomal-recessive NSHL, accounting for 80.42% (184/226) of the genetic basis in all hereditary hearing loss patients. The genetic spectrum for autosomal-dominant NSHL seems to be wide and not focused on one or a few genes; this result differs from the findings of a study involving Japanese subjects, which reported *KCNQ4* as the most frequent cause for dominant NSHL [31]. We made a comparison across multiple populations (Table 4) and found the genetic diagnostic rate varied from 12.7 to 66.7% which may due to the number of genes captured, variations in patient selection criteria, previous genetic testing and enrollment case numbers [33–46]. In the cases with Caucasian ethnicity, nearly three-fourths of all diagnoses were attributable to ten genes and the four genes most frequently implicated were *GJB2* (22%), *STRC* (16%), *SLC26A4* (7%), and *TECTA* (5%), which is different from those of our study except *GJB2*. The pathogenesis of 433 Chinese sporadic hearing loss patients was shown in Fig. 3a. The ratio of hereditary factors in congenital hearing loss is at least 60%, and the cause of the other 40% was considered to be environmental and unknown factors [2, 3]. After DA3 testing, there are 7.81% of the SNHL patients in this study assumed to be due to genetic factors yet to be discovered. A comparison of the positive diagnostic rate in the common deafness genes (*GJB2*, *SLC26A4*, *and 12S rRNA*) in this case cohort with that in a larger sample (16,456 cases) from our deafness genetic testing center revealed that our sample selection was representative of the Chinese deafness population (Fig. 3b). Although targeted genomic enrichment followed by NGS has been shown to be an efficient strategy for the clinical diagnosis of hearing loss, in the Chinese Han population, a first-line test for frequent genes with Sanger sequencing is advantageous for economic and time-saving considerations.

**Table 4:** Comparisons of the molecular diagnostic rate of reported NGS studies on hearing loss

	No. of genes tested	CNVs analysis	Pre-genetic testing	Exclusion of positive cases of pre-testing	Ethnicity	No. of cases	Diagnostic rate	Pedigrees or cases with clear family history	Diagnostic rate	Study
1	246	No	*GJB2*	Yes	Israeli Jewish and Palestinian Arab			11	55%	Brownstein et al. [45]
2	54	Yes	No		Mixed	100	42%			Shearer et al. [33]
3	79	No	*GJB2, SLC26A4,* and *MT-RNR1*	Yes	Han Chinese	93	20.47%	32	43.75%	Yang et al. [43]
4	80	No	20 common variants in *GJB2, SLC26A4,* and *MT-RNR1*	Yes	Han Chinese			12	33.33%	Wu et al. [42]
5	84	No	*GJB2,OTOF, Mitochondrial* 1555A>G or 3243A>G	Yes	Japanese			15	46.67%	Mutai et al. [41]
6	96	No	No		Italy and Qatar			12	33.33%	Vozzi et al. [44]
7	131	Yes	*GJB2, SLC26A4,* and *MT-RNR1*	Yes	Chinese	63	12.7%			Gu et al. [51]
8	66 or 89	Yes	No		Mixed	686	38.9%			Shearer et al. [17, 34]
9	104 and 3 microRNA regions	No	Nine hotspot mutations of *GJB2, SLC26A4, GJB3, MT-RNR1*	No	Chinese			23	30.43%	Wei et al. [36]
10	204	Yes	Phenotype driven candidate gene testing and *GJB2*	Yes	Korea	53	20.75%			Park et al. [39]
11	80 or 129	Yes prescreen	*GJB2*	No	Mainly European			23	57%	Vona et al. [37]
12	97	No	*GJB2, SLC26A4* and *MT-RNR1*	Yes	Uyghur Chinese			6	66.7%	Chen et al. [46]
13	66 or 89	Yes	No		Mixed	604	37%	376	41% (AR) 50% (AD)	Sloan-Heggen et al. [15]
14	WES followed by targeted analysis of 120 genes	Yes	No		Dutch	200	33.5%			Zazo Seco et al. [40]
15	Clinical exome sequencing of 4813 genes	No	No		Caucasian origin	49 (including 32 from the nonsyndromic, non-GJB2 group and 17 from the syndromic group)	21% (nonsyndromic, non-GJB2 group) 47% (syndromic group)			Likar et al. [38]
16	119 + Mitochrondrial genome	No	*GJB2, SLC26A4* and *MT-RNR1*	No	Chinese	433	52.19%	30	56.67%	This study

### The importance of ethnically matched MAF filtering

Extensive optimization and evaluation procedures are required for all NGS platforms to ensure a reliable and routine application of NGS technologies in diagnostics. We included a filtering step based on NGS data of MAFs in 616 Chinese normal hearing controls to minimize false-positive results [20]. In aggregate, ethnicity-specific MAF filtering helped reduce the list of VUS variants from 6184 to 5643. This step improved the annotation of identified variants and allowed us to recategorize 541 variants as benign that were otherwise annotated as VUS, and recategorize several variants as VUS that were otherwise annotated as pathogenic by the 1000 Genomes, ESP, ExAC, HapMap, and Wellderly databases.

The reported *GJB3* digenic variants c.580G>A and the dominant variant c.538C>T appeared to be nonpathogenic. In our study, six patients and six controls were *GJB3* c.580G>A heterozygotes. None of the six deafness patients carried the pathogenic *GJB2* variants reported by Liu et al. [47]. Three patients and one control were *GJB3* c.538C>T heterozygotes. Considering the Sanger results and phenotype of family members, we recategorized c.580G>A (BA1) and c.538C>T (BS1 + BS4) as benign. Our study supports the findings that querified variants in *GJB3* as a cause of deafness [17]. Further studies are required to verify whether *GJB3* variants cause fully penetrant deafness.

### Molecular diagnosis in patients with SNHL and inner ear malformations

The etiologies of inner ear malformations have not been completely clarified. Genotype and phenotype correlations were determined in EVA and IP-III. In this study, we observed at least seven types of inner ear malformations in 100 patients; the rate of inner ear malformations in SNHL was 23.09% (100/433). The most common type was EVA (89 cases), which is an autosomal-recessive disease. In our study, 94.3% (84/89) of EVA cases carried bi-allelic causative variants, 3.37% (3/89) had monoallelic, and 2.25% (2/89) had no variant in *SLC26A4*. Neither *KCNJ10* nor *FOXI I* variants contributed to the molecular etiology of EVA among patients in this study. The relevance ratio of bi-allelic *SLC26A4* variants is 36% in Caucasians, 66% in Japanese, and 81% in Koreans [48–50]. In this study, the second most common type was IP-III, an X-linked genetic disease in which the basal turn of the cochlea is placed directly at the end of the inner auditory canal (three cases); 100% of the male patients with this malformation carried hemizygous variants in *POU3F4*. Other inner ear malformations included cochlea aplasia (one case), cochlea hypoplasia (one case), isolated Mondini deformity (three cases), common cavity deformity (two cases), and narrow internal auditory canal (one case). No genetic pathogenesis was identified in the types of inner ear deformity other than EVA and IP-III, indicating that the etiology of inner ear malformation may be multifactorial.

### Molecular diagnosis in patients with syndromic SNHL

The clinical diagnosis of syndromic SNHL helps direct the test panel choice, thereby saving time and cost. Three patients with Waardenburg syndrome (WS) were diagnosed clinically by the phenotype of hearing loss with dermal pigmentation and iris heterochrosis. In two of the WS cases, genetic variants in *PAX3* and *MITF* were identified, respectively, whereas the other case showed no causative variant in WS-related genes *(PAX3*, *MITF*, *SNAI2*, *and EDNRB)*. No Pendred syndrome was diagnosed in our patient cohort, which may be due to the fact that the average age of our patients with EVA was 9.36 years. The phenotype of goiter is not present at birth, developing in early puberty (40%) or adulthood (60%). In addition, it is possible that our sample size was not large enough. We identified ten cases with bi-allelic variants in the Usher syndrome-related genes, *USH2A*, *MYO7A*, *CDH23,* and *ADGRV1*. Usher syndrome is a clinically and genetically heterogeneous autosomal-recessive disorder characterized by sensorineural hearing deficiencies and later development of progressive retinitis pigmentosa. It is the most frequent cause of combined deafness and blindness in adults and affects 3–6% of children born with hearing impairment. None of the ten cases had vestibular dysfunction or hypoplastic, and vestibular and ophthalmologic examinations were not performed at the beginning of this study. The average age of these ten patients was 12.77 years. Hearing loss occurred postlingually in five cases, prelingually in four, and one with ambiguous onset. In consideration of the variable extent of vestibular involvement and the usual onset of retinitis pigmentosa symptoms in the second decade of life, vestibular evaluation and ophthalmologic follow-up will be performed in the future. In addition, there is the possibility that variants in *MYO7A* and *CDH23* caused DFNB2 and DFNB12.

## Conclusions

We demonstrated that the DA3 panel assay covering 129 genes had a positive diagnostic rate of 52.19% on sequence alterations for sporadic SNHL patients and 56.67% for deafness pedigrees. WES or WGS should be reserved only for negative cases as an opportunity to discover novel candidate genes. Ethnically matched MAF filtering changed the categorization of 8.75% of our list of variants from VUS to benign. This report highlight the clinical utility of NGS panels identifying disease-causing variants for the clinical diagnosis of deafness and underlines the importance of a broad data of control and the ACMG/AMP guidelines for accurate clinical delineation of VUS variants. The results provide more detailed information for genetic consulting to predict the risk of recurrence.

## Supplementary information

Tables S1–S4

Table S5

## Data Availability

All data are presented in the manuscript or additional files.
