# Supplementary material for: Comprehensive genetic testing of Chinese SNHL patients and variants interpretation using ACMG guidelines and ethnically matched normal controls
Source: Eur J Hum Genet. 2019 Sep 20;28(2):231–43. doi: 10.1038/s41431-019-0510-6 (PMC6974605; doi:10.1038/s41431-019-0510-6)
Supplement: Supplementary file 1 — Tables S1–S4 [file 41431_2019_510_MOESM1_ESM.docx]

**Table S1 Deafness V3 List**

| **Gene** | | **NCBI** | **OMIM** | **Description** | **CDS** | **No. of** | **Covered** | **Inheritance pattern** | | | | **Syndrome** |
| --- | --- | --- | --- | --- | --- | --- | --- | --- | --- | --- | --- | --- |
|  | | **Reference** |  |  | **bps** | **Exons** | **Region** | **AD*** | **AR*** | | **X*** |  |
| *ACTB* | NM_001101.3 | | 102630 | Actinβ | 1852 | 6 | CDS only | √ | |  |  | √ |
| *ACTG1* | NM_001199954.1 | | 102560 | Actinγ1 | 2123 | 6 | CDS only | √ | |  |  | √ |
| *ATP6V1B1* | NM_001692.3 | | 192132 | Lysosomal ATPase, H+ transporting, V1 subunit B1 | 1956 | 14 | CDS only |  | | √ |  |  |
| *ATP6V1B2* | NM_001693.3 | | 606939 | Lysosomal ATPase, H+ transporting, V1 subunit B2 | 3054 | 14 | CDS only | √ | |  |  | √ |
| *BCS1L* | NM_001079866.1 | | 603647 | Ubiquinol-cytochrome c reductase synthesis-like | 1454 | 8 | CDS only |  | | √ |  | √ |
| *BSND* | NM_057176.2 | | 606412 | Bartter syndrome, infantile, with sensorineural deafness | 1396 | 4 | CDS only |  | | √ |  | √ |
| *CATSPER2* | NM_001282310.1 | | 607249 | Cation channel, sperm associated 2 | 1755 | 13 | CDS only |  | | √ |  |  |
| *CCDC50* | NM_178335.2 | | 611051 | Coiled-coil domain containing 50 | 8949 | 12 | CDS only | √ | |  |  |  |
| *CDH23* | NM_022124.5 | | 605516 | Cadherin-related 23 | 11134 | 70 | CDS only |  | | √ |  | √ |
| *CEACAM16* | NM_001039213.2 | | 614591 | Carcinoembryonic antigen-related cell adhesion molecule 16 | 1692 | 7 | CDS only | √ | |  |  |  |
| *CLDN14* | NM_001146077.1 | | 605608 | Claudin 14 | 1469 | 3 | CDS only |  | | √ |  |  |
| *CLRN1* | NM_174878.2 | | 606397 | Clarin 1 | 2359 | 8 | CDS only |  | | √ |  | √ |
| *COCH* | NM_001135058.1 | | 603196 | Cochlin | 2882 | 11 | CDS only | √ | |  |  |  |
| *COL11A2* | NM_080680.2 | | 120290 | Collagen, type XI,α2 | 6425 | 66 | CDS only | √ | | √ |  | √ |
| *COL9A2* | NM_001852.3 | | 120260 | Collagen, type IX,α2 | 2831 | 32 | CDS only | √ | | √ |  | √ |
| *COL9A3* | NM_001853.3 | | 120270 | Collagen, type IX,α3 | 2485 | 32 | CDS only | √ | |  |  |  |
| *CRYM* | NM_001888.3 | | 123740 | Crystallin, mu | 1506 | 10 | CDS only | √ | |  |  |  |
| *GSDME* | NM_004403.2 | | 608798 | Deafness, autosomal dominant 5 | 2521 | 10 | CDS only | √ | |  |  |  |
| *WHRN* | NM_001173425.1 | | 607928 | Deafness, autosomal recessive 31 (Whirlin) | 4076 | 12 | CDS only |  | | √ |  |  |
| *PJVK* | NM_001042702.3 | | 610219 | Deafness, autosomal recessive 59 | 1534 | 7 | CDS only |  | | √ |  |  |
| *DIAPH1* | NM_005219.4 | | 602121 | Diaphanous-related formin 1 | 5804 | 28 | CDS only | √ | | √ |  | √ |
| *DSPP* | NM_014208.3 | | 125485 | Dentin sialophosphoprotein | 4331 | 5 | CDS only | √ | |  |  |  |
| *ECE1* | NM_001397.2 | | 600423 | Endothelin converting enzyme 1 | 5114 | 19 | CDS only | √ | |  |  |  |
| *EDNRA* | NM_001957.3 | | 131243 | Endothelin receptor type A | 4168 | 8 | CDS only | √ | |  |  |  |
| *EDNRB* | NM_000115.3 | | 131244 | Endothelin receptor type B | 4296 | 8 | CDS only | √ | | √ |  | √ |
| *ERCC2* | NM_000400.3 | | 126340 | Excision repair cross-complementing | 2568 | 23 | CDS only |  | | √ |  | √ |
|  |  | |  | rodent repair deficiency, | | |  |  | |  |  |  |
|  |  | |  | complementation group 2 | | |  |  | |  |  |  |
| *ERCC3* | NM_000122.1 | | 133510 | Excision repair cross-complementing | 2751 | 15 | CDS only |  | | √ |  | √ |
|  |  | |  | rodent repair deficiency, | | |  |  | |  |  |  |
|  |  | |  | complementation group 3 | | |  |  | |  |  |  |
| *ESPN* | NM_031475.2 | | 606351 | Espin | 3531 | 13 | CDS only |  | | √ |  |  |
| *ESRRB* | NM_004452.3 | | 602167 | Estrogen-related receptorβb | 3029 | 11 | CDS only |  | | √ |  |  |
| *EYA4* | NM_004100.4 | | 603550 | Eyes absent homolog 4 | 5697 | 20 | CDS only | √ | |  |  |  |
| *FAS* | NM_000043.4 | | 134637 | Fas cell surface death receptor | 2755 | 9 | CDS only | √ | |  |  | √ |
| *FGF3* | NM_005247.2 | | 164950 | Fibroblast growth factor 3 | 1548 | 3 | CDS only |  | | √ |  | √ |
| *FGFR3* | NM_000142.4 | | 134934 | Fibroblast growth factor receptor 3 | 4304 | 18 | CDS only | √ | | √ |  | √ |
| *FOXI1* | NM_012188.4 | | 601093 | Forkhead box I1 | 2296 | 2 | CDS only |  | | √ |  |  |
| *GATA3* | NM_001002295.1 | | 131320 | GATA binding protein 3 | 3070 | 6 | CDS only | √ | |  |  |  |
| *GIPC3* | NM_133261.2 | | 608792 | GIPC PDZ domain containing family, member 3 | 4317 | 6 | CDS only |  | | √ |  |  |
| *GJA1* | NM_000165.3 | | 121014 | Gap junction protein,α1 | 3130 | 2 | CDS only | √ | | √ |  | √ |
| *GJB1* | NM_000166.5 | | 304040 | Gap junction protein,β1 | 1674 | 2 | CDS only |  | |  | √ |  |
| *GJB2* | NM_004004.5 | | 121011 | Gap junction protein,β2 | 2347 | 3 | Entire gene + 5kb upstream | √ | | √ |  | √ |
| *GJB3* | NM_001005752.1 | | 603324 | Gap junction protein,β3 | 1777 | 2 | CDS only | √ | |  |  |  |
| *GJB4* | NM_153212.2 | | 605425 | Gap junction protein,β4 | 2840 | 2 | CDS only | √ | | √ |  |  |
| *GJB6* | NM_001110219.2 | | 604418 | Gap junction protein,β6 | 2178 | 5 | CDS only | √ | | √ |  | √ |
| *ADGRV1* | NM_032119.3 | | 602851 | G protein-coupled receptor 98 | 19333 | 90 | CDS only | √ | | √ |  | √ |
| *GPSM2* | NM_013296.4 | | 609245 | G-protein signaling modulator 2 | 3039 | 15 | CDS only |  | | √ |  | √ |
| *GRHL2* | NM_024915.3 | | 608576 | Grainyhead-like 2 | 5231 | 16 | CDS only | √ | | √ |  | √ |
| *GRXCR1* | NM_001080476.2 | | 613283 | Glutaredoxin, cysteine rich 1 | 1003 | 4 | CDS only |  | | √ |  |  |
| *GSTP1* | NM_000852.3 | | 134660 | Glutathione S-transferaseπ1 | 986 | 7 | CDS only | - | | - | - | - |
| *HAL* | NM_002108.3 | | 609457 | Histidine ammonia-lyase | 3927 | 21 | CDS only | √ | |  |  |  |
| *HGF* | NM_000601.4 | | 142409 | Hepatocyte growth factor | 2820 | 18 | CDS only |  | | √ |  |  |
| *ILDR1* | NM_001199799.1 | | 609739 | Immunoglobulin-like domain containing receptor 1 | 2908 | 8 | CDS only |  | | √ |  |  |
| *JAG1* | NM_000214.2 | | 601920 | Jagged 1 | 5988 | 26 | CDS only |  | | √ |  | √ |
| *KCNE1* | NM_000219.4 | | 176261 | Potassium voltage-gated channel, Isk-related family, member 1 | 3575 | 4 | CDS only | √ | | √ |  | √ |
| *KCNJ10* | NM_002241.4 | | 602208 | Potassium inwardly-rectifying channel, subfamily J, member 10 | 5323 | 2 | CDS only | √ | | √ |  | √ |
| *KCNQ1* | NM_000218.2 | | 607542 | Potassium voltage-gated channel, KQT-like subfamily, member 1 | 3262 | 16 | CDS only | √ | | √ |  | √ |
| *KCNQ4* | NM_004700.3 | | 603537 | Potassium voltage-gated channel, KQT-like subfamily, member 4 | 4116 | 14 | CDS only | √ | |  |  |  |
| *CEMIP* | NM_018689.1 | | 608366 | Cell migration inducing hyaluronidase 1 | 7080 | 29 | CDS only | - | | - | - | - |
| *LHFPL5* | NM_182548.3 | | 609427 | Lipoma HMGIC fusion partner-like 5 | 2147 | 4 | CDS only |  | | √ |  |  |
| *LHX3* | NM_014564.3 | | 600577 | LIM homeobox 3 | 2419 | 6 | CDS only |  | | √ |  |  |
| *LOXHD1* | NM_144612.6 | | 613072 | Lipoxygenase homology domains 1 | 6854 | 40 | Chr18: |  | | √ |  |  |
|  |  | |  |  |  |  | 44152049- |  | |  |  |  |
|  |  | |  |  |  |  | 44152125 |  | |  |  |  |
| *LRTOMT* | NM_001145309.3 | | 612414 | Leucine rich transmembrane and O-methyltransferase domain containing | 3844 | 9 | CDS only |  | | √ |  |  |
| *MARVELD2* | NM_001038603.2 | | 610572 | MARVEL domain containing 2 | 2297 | 7 | CDS only |  | | √ |  |  |
| *mir182* | NR_029614.1 | | 611607 | microRNA 182 | 110 | 1 | CDS only | - | | - | - | - |
| *mir183* | NR_029615.1 | | 611608 | microRNA 183 | 110 | 1 | CDS only | - | | - | - | - |
| *mir96* | NR_029512.1 | | 611606 | microRNA 96 | 78 | 1 | CDS only | √ | |  |  |  |
| *MITF* | NM_198159.2 | | 156845 | Microphthalmia-associated transcription factor | 4815 | 10 | CDS only | √ | | √ |  | √ |
| *MSRB3* | NM_198080.3 | | 613719 | Methionine sulfoxide reductase B3 | 4307 | 6 | CDS only |  | | √ |  |  |
| *MTAP* | NM_002451.3 | | 156540 | Methylthioadenosine phosphorylase | 4937 | 8 | CDS only | √ | |  |  |  |
| *MT-TD* | NC_012920.1 | | 590015 | Mitochondrial transfer RNA aspartic acid | 68 | 1 | CDS only | - | | - | - | - |
| *MT-TH* | NC_012920.1 | | 590040 | Mitochondrial transfer RNA histidine | 69 | 1 | CDS only | - | | - | - | - |
| *MT-TI* | NC_012920.1 | | 590045 | Mitochondrial transfer RNA isoleucine | 69 | 1 | CDS only | - | | - | - | - |
| *MT-TK* | NC_012920.1 | | 590060 | Mitochondrial transfer RNA lysine | 70 | 1 | CDS only | - | | - | - | - |
| *MT-TL1* | NC_012920.1 | | 590050 | Mitochondrial transfer RNA leucine | 75 | 1 | CDS only | - | | - | - | - |
| *MT-L2* | NC_012920.1 | | 590055 | Mitochondrial transfer RNA leucine2 | 71 | 1 | CDS only | - | | - | - | - |
| *MT-TM* | NC_012920.1 | | 590065 | Mitochondrial transfer RNA methionine | 68 | 1 | CDS only | - | | - | - | - |
| *MT-TQ* | NC_012920.1 | | 590030 | Mitochondrial transfer RNA glutamine | 72 | 1 | CDS only | - | | - | - | - |
| *MT-TS1* | NC_012920.1 | | 590080 | Mitochondrial transfer RNA serine 1 | 69 | 1 | CDS only | - | | - | - | - |
| *MT-TS2* | NC_012920.1 | | 590085 | Mitochondrial transfer RNA serine 2 | 59 | 1 | CDS only | - | | - | - | - |
| *MYH14* | NM_001145809.1 | | 608568 | Myosin, heavy chain 14, non-muscle | 6930 | 43 | CDS only | √ | |  |  |  |
| *MYH9* | NM_002473.4 | | 160775 | Myosin, heavy chain 9, non-muscle | 7505 | 41 | CDS only | √ | |  |  | √ |
| *MYO15A* | NM_016239.3 | | 602666 | Myosin XVA | 11876 | 66 | CDS only |  | | √ |  |  |
| *MYO1A* | NM_001256041.1 | | 601478 | Myosin IA | 3658 | 29 | CDS only | √ | |  |  |  |
| *MYO1C* | NM_001080779.1 | | 606538 | Myosin IC | 4973 | 32 | CDS only | - | | - | - | - |
| *MYO1F* | NM_012335.3 | | 601480 | Myosin IF | 4173 | 28 | CDS only | - | | - | - | - |
| *MYO3A* | NM_017433.4 | | 606808 | Myosin IIIA | 5798 | 35 | CDS only |  | | √ |  |  |
| *MYO6* | NM_004999.3 | | 600970 | Myosin IV | 8662 | 35 | CDS only | √ | | √ |  |  |
| *MYO7A* | NM_000260.3 | | 276903 | Myosin 7A | 7465 | 49 | CDS only | √ | | √ |  | √ |
| *NDP* | NM_000266.3 | | 300658 | Norrie disease | 2058 | 3 | CDS only |  | |  | √ |  |
| *NR2F1* | NM_005654.5 | | 132890 | Nuclear receptor subfamily 2, group F, member 1 | 1983 | ND | CDS only | √ | |  |  | √ |
| *OTOA* | NM_144672.3 | | 607038 | Otoancorin | 3624 | 28 | CDS only |  | | √ |  |  |
| *OTOF* | NM_194248.2 | | 603681 | Otoferlin | 7171 | 47 | CDS only |  | | √ |  |  |
| *OTOR* | NM_020157.3 | | 606067 | Otoraplin | 1482 | 4 | CDS only | - | | - | - | - |
| *P2RX2* | NM_170683.3 | | 600844 | Purinergic receptor P2X, ligand-gated ion channel,2 | 1945 | 10 | CDS only | √ | |  |  |  |
| *PAX3* | NM_181457.3 | | 606597 | Paired box 3 | 2032 | 8 | CDS only | √ | | √ |  | √ |
| *PCDH15* | NM_001142763.1 | | 605514 | Protocadherin-related 15 | 7042 | 33 | CDS only |  | | √ |  | √ |
| *PDZD7* | NM_001195263.1 | | 612971 | PDZ domain-containing 7 | 4164 | 17 | CDS only |  | | √ |  | √ |
| *PMP22* | NM_000304.3 | | 601097 | Peripheral myelin protein 22 | 1861 | 5 | CDS only | √ | | √ |  | √ |
| *POU3F4* | NM_000307.4 | | 300039 | POU class 3 homeobox 4 | 1507 | 1 | CDS only |  | |  | √ |  |
| *POU4F3* | NM_002700.2 | | 602460 | POU class 4 homeobox 3 | 1182 | 2 | CDS only | √ | |  |  |  |
| *PRPS1* | NM_002764.3 | | 311850 | Phosphoribosyl pyrophosphate synthetase 1 | 2156 | 7 | CDS only |  | |  | √ | √ |
| *PTPRR* | NM_001145026.1 | | 603317 | Protein tyrosine phosphatase, receptor type, Q | 8066 | 3 | Chr12:  80849273-80849842 80878212-80878383 |  | | √ |  |  |
| *RDX* | NM_001260492.1 | | 179410 | Radixin | 2761 | 14 | CDS only |  | | √ |  |  |
| *SERPINB6* | NM_004568.5 | | 173321 | Serpin peptidase inhibitor, clade B (ovalbumin), member 6 | 1932 | 7 | CDS only |  | | √ |  |  |
| *SIX1* | NM_005982.3 | | 601205 | SIX homeobox 1 | 2687 | 2 | CDS only | √ | |  |  | √ |
| *SIX5* | NM_175875.4 | | 600963 | SIX homeobox 5 | 3352 | 3 | CDS only | √ | |  |  | √ |
| *SLC17A8* | NM_139319.2 | | 607557 | Solute carrier family 17 (vesicular glutamate transporter), member 8 | 3983 | 12 | CDS only | √ | |  |  |  |
| *SLC26A4* | NM_000441.1 | | 605646 | Solute carrier family 26 (anion exchanger), member 4 | 4930 | 21 | CDS only |  | | √ |  | √ |
| *SLC26A5* | NM_198999.2 | | 604943 | Solute carrier family 26 (anion exchanger), member 5 | 2697 | 20 | CDS only |  | | √ |  |  |
| *SLC4A11* | NM_001174090.1 | | 610206 | Solute carrier family 4, sodium borate transporter, member 11 | 3268 | 20 | CDS only | √ | | √ |  |  |
| *SMPX* | NM_014332.2 | | 300226 | Small muscle protein, X-linked | 951 | 5 | CDS only |  | |  | √ |  |
| *SNAI2* | NM_003068.4 | | 602150 | Snail family zinc finger 2 | 2112 | 3 | CDS only | √ | |  |  | √ |
| *SOX2* | NM_003106.3 | | 184429 | SRY (sex determining region Y)-box 2 | 2520 | 1 | CDS only | √ | |  |  | √ |
| *SPINK5* | NM_001127698.1 | | 605010 | Serine peptidase inhibitor, Kazal type 5 | 3745 | 34 | CDS only | √ | | √ |  | √ |
| *STRC* | NM_153700.2 | | 606440 | Stereocilin | 5515 | 29 | CDS only |  | | √ |  |  |
| *TBL1X* | NM_005647.3 | | 300196 | Transducin (β)-like 1X-linked | 5715 | 18 | CDS only | - | | - | √ | - |
| *TCF21* | NM_198392.2 | | 603306 | Transcription factor 21 | 3249 | 3 | CDS only | - | | - | - | - |
| *TECTA* | NM_005422.2 | | 602574 | Tectorinα a | 6468 | 23 | CDS only | √ | | √ |  |  |
| *TFCP2* | NM_005653.4 | | 189889 | Transcription factor CP2 | 3715 | 15 | CDS only | √ | | - | - | - |
| *TIMM8A* | NM_004085.3 | | 300356 | Translocase of inner mitochondrial membrane 8 homolog A | 1459 | 2 | CDS only |  | |  | √ | √ |
| *TJP2* | NM_004817.3 | | 607709 | Tight junction protein 2 | 4725 | 23 | CDS only |  | | √ |  |  |
| *TMC1* | NM_138691.2 | | 606706 | Transmembrane channel-like 1 | 3201 | 24 | CDS only | √ | | √ |  |  |
| *TMIE* | NM_147196.2 | | 607237 | Transmembrane inner ear | 1861 | 4 | CDS only |  | | √ |  |  |
| *TMPRSS3* | NM_024022.2 | | 605511 | Transmembrane protease, serine 3 | 2463 | 13 | CDS only |  | | √ |  |  |
| *TMPRSS5* | NM_030770.2 | | 606751 | Transmembrane protease, serine 5 | 2233 | 13 | CDS only | - | | √ | - | - |
| *TPRN* | NM_001128228.2 | | 613354 | Taperin | 2641 | 4 | CDS only |  | | √ |  |  |
| *TRIOBP* | NM_001039141.2 | | 609761 | TRIO and F-actin binding protein | 10159 | 24 | CDS only |  | | √ |  |  |
| *USH1C* | NM_153676.3 | | 605242 | Usher syndrom 1C | 3246 | 28 | CDS only |  | | √ |  | √ |
| *USH1G* | NM_173477.3 | | 607696 | Usher syndrome 1G | 3568 | 3 | CDS only |  | | √ |  | √ |
| *USH2A* | NM_206933.2 | | 608400 | Usher syndrome 2A | 18883 | 72 | CDS only |  | | √ |  | √ |
| *WFS1* | NM_006005.3 | | 606201 | Wolfram syndrome 1 | 3640 | 8 | CDS only | √ | | √ |  | √ |
|  | |  |  |  |  |  |  |  | |  |  |  |

**Table S2 Pathogenic and likely pathogenic variants in 119 deafness genes**

| **Variants classification** | **HGNC** | **Chromosome Position** | **HGVSc** | **HGVSp** |
| --- | --- | --- | --- | --- |
| PVS1+PM2+PP3 | *CDH23* | 73544043 | [NG_008835.1](https://www.ncbi.nlm.nih.gov/projects/sviewer/?id=NG_008835.1&search=NG_008835.1:g.283185G%3EA&v=1:100&content=5)([NM_022124.5](https://www.ncbi.nlm.nih.gov/clinvar/variation/162864/)):c.5369-1G>T | . |
| PVS1+PM2+PP3 | *CDH23* | 73485278 | [NG_008835.1](https://www.ncbi.nlm.nih.gov/projects/sviewer/?id=NG_008835.1&search=NG_008835.1:g.283185G%3EA&v=1:100&content=5)([NM_022124.5](https://www.ncbi.nlm.nih.gov/clinvar/variation/162864/)):c.3579+1G>A | . |
| PVS1+PM2+PP3 | *CDH23* | 73569774 | [NM_022124.5](https://www.ncbi.nlm.nih.gov/clinvar/variation/162864/):c.8920G>T | NP_071407.4:p.(Glu2974*) |
| PVS1+PM2+PP3 | *WHRN* | 117267056 | NM_015404.3:c.26C>A | NP_056219.3:p.(Ser9*) |
| PVS1+PM2+PP3 | *GIPC3* | 3589847 | NM_133261.2:c.724G>T | NP_573568.1:p.(Glu242*) |
| PVS1+PS4+PM3 | *GJB2* | 20763420 | NM_004004.5:c.299_300delAT | NP_003995.2:p.(His100Argfs*14) |
| PVS1+PS4+PM3 | *GJB2* | 20763529 | NM_004004.5:c.176_191delGCTGCAAGAACGTGTG | NP_003995.2:p.(Gly59Alafs*18) |
| PVS1+PS4+PM2+PM3 | *GJB2* | 20763209 | NM_004004.5:c.508_511dupAACG | NP_003995.2:p.(Ala171Glufs*40) |
| PVS1+PS4+PP3+PM3 | *GJB2* | 20763485 | NM_004004.5:c.235delC | NP_003995.2:p.(Leu79Cysfs*3) |
| PVS1+PM2+PM3 | *GJB2* | 20766921 | [NG_008358.1](https://www.ncbi.nlm.nih.gov/projects/sviewer/?id=NG_008358.1&search=NG_008358.1:g.10227G%3EA&v=1:100&content=5)(NM_004004.5):c.-23+1G>A | . |
| PVS1+PM2+PM3+PP3 | *GJB2* | 20763712 | NM_004004.5:c.9G>A | NP_003995.2:p.(Trp3*) |
| PVS1+PM2+PP3 | *ERCC2* | 45872251 | [NG_007067.2](https://www.ncbi.nlm.nih.gov/projects/sviewer/?id=NG_007067.2&search=NG_007067.2:g.13844G%3ET&v=1:100&content=5)([NM_000400.3](https://www.ncbi.nlm.nih.gov/projects/sviewer/?id=NM_000400.3&search=NM_000400.3:c.468A%3EC&v=1:100&content=5)):c.184-1G>T | . |
| PVS1+PM2+PP3 | *ERCC2* | 45871950 | [NM_000400.3](https://www.ncbi.nlm.nih.gov/projects/sviewer/?id=NM_000400.3&search=NM_000400.3:c.468A%3EC&v=1:100&content=5):c.298G>T | NP_000391.1:p.(Glu100*) |
| PVS1+PM2+PP3 | *ERCC2* | 45872390 | [NM_000400.3](https://www.ncbi.nlm.nih.gov/projects/sviewer/?id=NM_000400.3&search=NM_000400.3:c.468A%3EC&v=1:100&content=5):c.121G>T | NP_000391.1:p.(Glu41*) |
| PVS1+PM2+PP3 | *ERCC3* | 128047339 | NM_000122.1:c.583C>T | NP_000113.1:p.(Arg195*) |
| PVS1+PS3+PM3 | *GJB2* | 20763685 | NM_004004.5:c.35dupG | NP_003995.2:p.(Val13Cysfs*35) |
| PVS1+PS3+PM3 | *GJB2* | 20763685 | NM_004004.5:c.35delG | NP_003995.2:p.(Gly12Valfs*2) |
| PVS1+PM2+PM3 | *GJB2* | 20763115 | NM_004004.5:c.560_605dupAGAAGACTGTCTTCACAGTGTTCATGATTGCAGTGTCTGGAATTTG | NP_003995.2:p.(Cys202*) |
| PVS1+PM2+PP3 | *GJB3* | 35250371 | NM_001005752.1:c.8G>A | NP_001005752.1:p.(Trp3*) |
| PVS1+PM2+PP3 | *ADGRV1* | 89938532 | NM_032119.3:c.2320G>T | NP_115495.3:p.(Gly774*) |
| PVS1+PM2+PP3 | *ADGRV1* | 90111471 | NM_032119.3:c.16114G>T | NP_115495.3:p.(Glu5372*) |
| PVS1+PM2+PP3 | *ADGRV1* | 90144634 | NM_032119.3:c.17200G>T | NP_115495.3:p.(Glu5734*) |
| PVS1+PM2+PP3 | *ADGRV1* | 89990458 | NM_032119.3:c.7885G>T | NP_115495.3:p.(Gly2629*) |
| PVS1+PM2+PP3 | *ILDR1* | 121720158 | NM_001199799.1:c.643G>T | NP_001186728.1:p.(Glu215*) |
| PVS1+PM2+PM3 | *SLC26A4* | 107323796 | NM_000441.1:c.915dupG | NP_000432.1:p.(Val306Glyfs*24) |
| PVS1+PM3+PP1 | *SLC26A4* | 107340599 | NM_000441.1:c.1686_1687insA | NP_000432.1:p.(Cys565Metfs*9) |
| PVS1+PM2+PM3 | *SLC26A4* | 107336458 | NM_000441.1:c.1520delT | NP_000432.1:p.(Leu507*) |
| PVS1+PM2+PM3 | *SLC26A4* | 107341583 | NM_000441.1:c.1746delG | NP_000432.1:p.(Ala584Argfs*2) |
| PVS1+PM2+PM3 | *SLC26A4* | 107334922 | NM_000441.1:c.1340delA | NP_000432.1:p.(Lys447Serfs*8) |
| PVS1+PM2+PM3 | *SLC26A4* | 107323801 | [NG_008489.1](https://www.ncbi.nlm.nih.gov/projects/sviewer/?id=NG_008489.1&search=NG_008489.1:g.61329G%3EC&v=1:100&content=5)([NM_000441.1](https://www.ncbi.nlm.nih.gov/projects/sviewer/?id=NM_000441.1&search=NM_000441.1:c.2218G%3EA&v=1:100&content=5)):c.918+2T>C | . |
| PVS1+PM2+PM3+PP3 | *SLC26A4* | 107302195 | NM_000441.1:c.109G>T | NP_000432.1:p.(Glu37*) |
| PVS1+PM2+PM3+PP3 | *SLC26A4* | 107334902 | NM_000441.1:c.1318A>T | NP_000432.1:p.(Lys440*) |
| PVS1+PM2+PM3+PP3 | *SLC26A4* | 107314782 | NM_000441.1:c.589G>T | NP_000432.1:p.(Gly197*) |
| PVS1+PM2+PM3+PP3 | *SLC26A4* | 107334923 | NM_000441.1:c.1339A>T | NP_000432.1:p.(Lys447*) |
| PVS1+PM2+PM3+PP3 | *SLC26A4* | 107340625 | [NG_008489.1](https://www.ncbi.nlm.nih.gov/projects/sviewer/?id=NG_008489.1&search=NG_008489.1:g.61329G%3EC&v=1:100&content=5)([NM_000441.1](https://www.ncbi.nlm.nih.gov/projects/sviewer/?id=NM_000441.1&search=NM_000441.1:c.2218G%3EA&v=1:100&content=5)): 1707+5G>A |  |
| PVS1+PM2+PP3 | *TECTA* | 121039501 | NM_005422.2:c.5866C>T | NP_005413.2:p.(Arg1956*) |
| PVS1+PM2+PP3 | *TMC1* | 75309494 | NM_138691.2:c.100C>T | NP_619636.2:p.(Arg34*) |
| PVS1+PM2+PP3 | *TMC1* | 75309631 | NG_008213.1(NM_138691.2):c.236+1G>A | . |
| PVS1+PM2+PP3 | *TRIOBP* | 38130634 | NM_001039141.2:c.4291G>T | NP_001034230.1:p.(Glu1431*) |
| PVS1+PM2+PP3 | *TRIOBP* | 38122087 | NM_001039141.2:c.3524C>A | NP_001034230.1:p.(Ser1175*) |
| PVS1+PM2+PP3 | *USH1C* | 17518361 | NG_011883.1(NM_005709.3):c.1591-1G>T | . |
| PVS1+PM2+PP3 | *USH1G* | 72915562 | NM_173477.4:c.1060G>T | NP_775748.2:p.(Glu354*) |
| PVS1+PM2+PP3 | *USH2A* | 216051224 | NM_206933.2:c.8559-2A>G | . |
| PVS1+PM3+PP1 | *USH2A* | 216380622 | NM_206933.2:c.3309C>A | NP_996816.2:p.(Tyr1103*) |
| PVS1+PM2+PP3 | *TMC1* | 75445556 | NM_138691.2:c.2218G>T | NP_619636.2:p.(Glu740*) |
| PVS1+PM2+PP3 | *TMPRSS3* | 43800225 | [NG_011629.1](https://www.ncbi.nlm.nih.gov/projects/sviewer/?id=NG_011629.1&search=NG_011629.1:g.11117G%3EA&v=1:100&content=5)([NM_024022.2](https://www.ncbi.nlm.nih.gov/clinvar/variation/46104/)):c.1048+1G>A | . |
| PVS1+PM2+PP3 | *TPRN* | 140095111 | NM_001128228.2:c.53G>A | NP_001121700.2:p.(Trp18*) |
| PVS1+PM2+PP3 | *MYO15A* | 18022591 | NM_016239.3:c.477G>A | NP_057323.3:p.(Trp159*) |
| PVS1+PM2+PP3 | *MYO15A* | 18059573 | NM_016239.3:c.8524G>T | NP_057323.3:p.(Glu2842*) |
| PVS1+PM2+PP3 | *MYO15A* | 18075054 | NM_016239.3:c.605C>A | NP_057323.3:p.(Ser202*) |
| PVS1+PM2+PP3 | *MYO15A* | 18036551 | NM_016239.3:c.4333G>T | NP_057323.3:p.(Glu1445*) |
| PVS1+PM2+PP3 | *MYO15A* | 18039064 | NM_016239.3:c.4522G>T | NP_057323.3:p.(Glu1508*) |
| PVS1+PM2+PP3 | *MYO15A* | 18039743 | NM_016239.3:c.4609G>T | NP_057323.3:p.(Glu1537*) |
| PVS1+PM2+PP3 | *MYO15A* | 18063303 | NM_016239.3:c9358C>T | NP_057323.3:p.(Gln3120*) |
| PVS1+PM2+PP3 | *MYO1A* | 57431768 | NM_005379.3:c.1846G>T | NP_005370.1:p.(Glu616*) |
| PVS1+PM2+PP3 | *MYO1A* | 57437892 | NM_005379.3:c.742C>T | NP_005370.1:p.(Gln248*) |
| PVS1+PM2+PP3 | *MYO3A* | 26385519 | NM_017433.4:c.1684A>T | NP_059129.3:p.(Arg562*) |
| PVS1+PM2+PP3 | *MYO3A* | 26385567 | NM_017433.4:c.1732G>T | NP_059129.3:p.(Glu578*) |
| PVS1+PM2+PP3 | *MYO7A* | 76886438 | NM_000260.3:c.2115C>A | NP_000251.3:p.(Cys705*) |
| PVS1+PM2+PP3 | *MYO7A* | 76888622 | NM_000260.3:c.2215G>T | NP_000251.3:p.(Glu739*) |
| PVS1+PM2+PP3 | *MYO7A* | 76872076 | NM_000260.3:c.1258A>T | NP_000251.3:p.(Lys420*) |
| PVS1+PM2+PP3 | *MYO7A* | 76900461 | NM_000260.3:c.3576G>A | NP_000251.3:p.(Trp1192*) |
| PVS1+PM2+PP3 | *MYO7A* | 76908641 | NM_000260.3:c.4439C>A | NP_000251.3:p.(Ser1480*) |
| PVS1+PM2+PP3 | *OTOF* | 26696342 | NM_194248.2:c.3502C>T | NP_919224.1:p.(Gln1168*) |
| PVS1+PM2+PP3 | *OTOF* | 26698784 | NM_194248.2:c.2989G>T | NP_919224.1:p.(Glu997*) |
| PVS1+PM2+PP3 | *OTOF* | 26703656 | NM_194248.2:c.1801G>T | NP_919224.1:p.(Glu601*) |
| PVS1+PM2+PP3 | *PCDH15* | 56424016 | NM_001142771.1:c.7C>T | NP_001136243.1:p.(Arg3*) |
| PVS1+PM2+PP3 | *PCDH15* | 55755492 | NM_001142771.1:c.2800C>T | NP_001136243.1:p.(Arg934*) |
| PVS1+PM2+PP3 | *POU3F4* | 82763502 | NM_000307.4:c.170G>A | NP_000298.3:p.(Trp57*) |
| PVS1+PS4+PM3 | *SLC26A4* | 107323898 | [NG_008489.1](https://www.ncbi.nlm.nih.gov/projects/sviewer/?id=NG_008489.1&search=NG_008489.1:g.61329G%3EC&v=1:100&content=5)([NM_000441.1](https://www.ncbi.nlm.nih.gov/projects/sviewer/?id=NM_000441.1&search=NM_000441.1:c.2218G%3EA&v=1:100&content=5)):c.919-2A>G | . |
| PVS1+PM2+PP3+PM3 | *SLC26A4* | 107344827 | NM_000441.1:c.2086C>T | NP_000432.1:p.(Gln696*) |
| PVS1+PM2+PP3+PM3 | *SLC26A4* | 107303811 | NM_000441.1:c.235C>T | NP_000432.1:p.(Arg79*) |
| PVS1+PM2+PM3 | *SLC26A4* | 107338557 | [NG_008489.1](https://www.ncbi.nlm.nih.gov/projects/sviewer/?id=NG_008489.1&search=NG_008489.1:g.61329G%3EC&v=1:100&content=5)([NM_000441.1](https://www.ncbi.nlm.nih.gov/projects/sviewer/?id=NM_000441.1&search=NM_000441.1:c.2218G%3EA&v=1:100&content=5)):c.1614+1G>A | . |
| PS1+PM2+PP1+PP3 | *CLDN14* | 37833300 | NM_001146077.1:c.694G>A | NP_001139549.1:p.(Gly232Arg) |
| PS1+PM2+PP1+PP3 | *ACTG1* | 79478250 | NM_001614.4:c.766C>T | NP_001605.1:p.(Arg256Trp) |
| PVS1+PM2 | *CDH23* | 73550904 | NM_022124.5:c.6065_6066insGTCA | NP_071407.4:p.(Gly2024Valfs*6) |
| PVS1+PM2 | *DSPP* | 88537062 | NM_014208.3:c.3248_3249insC | NP_055023.2:p.(Glu1083Aspfs*4) |
| PVS1+PM2 | *EDNRB* | 78492652 | NM_000115.3:c.57C>A | NP_000106.1:p.(Cys19*) |
| PVS1+PM2 | *FGF3* | 69625331 | NM_005247.2:c.462C>G | NP_005238.1:p.(Tyr154*) |
| PVS1+PM2 | *FGFR3* | 1808923 | NM_000142.4:c.2287C>T | [NP_001341738.1](https://www.ncbi.nlm.nih.gov/projects/sviewer/?id=NP_001341738.1&search=NP_001341738.1:p.Phe384Ile&v=1:100&content=5):p.(Arg763*) |
| PS3+PM3+PP1+PP3 | *GJB2* | 20763612 | NM_004004.5:c.109G>A | NP_003995.2:p.(Val37Ile) |
| PM2+PM3+PP1+PP3 | *GJB2* | 20763282 | NM_004004.5:c.439G>A | NP_003995.2:p.(Glu147Lys) |
| PM2+PM3+PP1+PP3 | *GJB2* | 20763294 | NM_004004.5:c.427C>T | NP_003995.2:p.(Arg143Trp) |
| PVS1+PM2 | *ADGRV1* | 90119241 | NG_007083.2([NM_032119.3](https://www.ncbi.nlm.nih.gov/projects/sviewer/?id=NM_032119.3&search=NM_032119.3:c.14905T%3EC&v=1:100&content=5)):c.161971G>T | . |
| PVS1+PM2 | *ADGRV1* | 90111486 | NM_032119.3:c.16129G>T | NP_115495.3:p.(Gly5377*) |
| P+PVS1+PM2 | *KCNQ1* | 2799253 | NM_000218.2:c.1780C>T | NP_000209.2:p.(Arg594*) |
| PS1+PM1+PM2+PP1+PP3 | *KCNQ4* | 41285852 | NM_004700.3:c.961G>A | NP_004691.2:p.(Gly321Ser) |
| PVS1+PM2 | *LRTOMT* | 71806088 | NM_001145308.4:c.383_384insGCTC | [NP_001138781.1](https://www.ncbi.nlm.nih.gov/projects/sviewer/?id=NP_001138781.1&search=NP_001138781.1:p.Arg208Gln&v=1:100&content=5):p.(Arg130Alafs*3) |
| PVS1+PM2 | *MARVELD2* | 68728503 | NG_017201.1(NM_001038603.2):c.1331+1G>A | . |
| PVS1+PM2 | *MITF* | 70005682 | NG_011631.1(NM_000248.3):c.710+1G>A | . |
| PVS1+PM2 | *MYO15A* | 18062238 | NG_011634.1(NM_016239.3):c.9084-1G>T | . |
| PVS1+PM2 | *MYO15A* | 18057211 | NG_011634.1(NM_016239.3):c.8088+1G>A | . |
| PVS1+PM2 | *MYO15A* | 18047315 | NG_011634.1(NM_016239.3):c.6177+1G>T | . |
| PVS1+PM2 | *MYO15A* | 18024085 | NM_016239.3:c.1971G>A | NP_057323.3:p.(Trp657*) |
| PVS1+PM2 | *MYO15A* | 18025347 | NM_016239.3:c.3233G>A | NP_057323.3:p.(Trp1078*) |
| PVS1+PM2 | *MYO15A* | 18046907 | NM_016239.3:c.5941G>T | NP_057323.3:p.(Glu1981*) |
| PVS1+PM2 | *MYO7A* | 76883793 | NG_009086.1(NM_000260.3):c.1798-1G>T | . |
| PVS1+PM2 | *MYO3A* | 26442778 | NG_011635.1(NM_017433.4):c.2636-1G>T | . |
| PVS1+PM2 | *MYO7A* | 76922383 | NG_009086.1(NM_000260.3):c.6237+1G>A | . |
| PVS1+PM2 | *OTOF* | 26690232 | NG_009937.1(NM_194248.2):c.4227+1G>T | . |
| PVS1+PM2 | *PCDH15* | 55721653 | NG_009191.3(NM_001142771.1):c.2884-1G>T | . |
| PS4+PM3+PP3 | *SLC26A4* | 107350577 | NM_000441.1:c.2168A>G | NP_000432.1:p.(His723Arg) |
| PS4+PM3+PP3 | *SLC26A4* | 107330593 | NM_000441.1:c.1174A>T | NP_000432.1:p.(Asn392Tyr) |
| PS4+PM2+PM3+PP3 | *SLC26A4* | 107330645 | NM_000441.1:c.1226G>A | NP_000432.1:p.(Arg409His) |
| PM2+PM3+PP1+PP3 | *SLC26A4* | 107329575 | NM_000441.1:c.1079C>T | NP_000432.1:p.(Ala360Val) |
| PS3+PM3+PP1+PP3 | *SLC26A4* | 107342443 | NM_000441.1:c.1975G>C | NP_000432.1:p.(Val659Leu) |
| PM2+PM3+PP1+PP3 | *SLC26A4* | 107314771 | NM_000441.1:c.578C>T | NP_000432.1:p.(Thr193Ile) |
| PM2+PM3+PP1+PP3 | *SLC26A4* | 107330648 | NM_000441.1:c.1229C>T | NP_000432.1:p.(Thr410Met) |
| PM2+PM3+PP1+PP3 | *SLC26A4* | 107302171 | NM_000441.1:c.85G>C | NP_000432.1:p.(Glu29Gln) |
| PS3+PM3+PP1+PP3 | *SLC26A4* | 107342495 | NM_000441.1:c.2027T>A | NP_000432.1:p.(Leu676Gln) |
| PM2+PM3+PP1+PP3 | *SLC26A4* | 107350571 | NM_000441.1:c.2162C>T | NP_000432.1:p.(Thr721Met) |
| PS3+PM3+PP1+PP3 | *SLC26A4* | 107303857 | NM_000441.1::c.281C>T | NP_000432.1:p.(Thr94Ile) |
| PM2+PM3+PP1+PP3 | *SLC26A4* | 107314782 | NM_000441.1:c.589G>A | NP_000432.1:p.(Gly197Arg) |
| PM2+PM3+PP1+PP3 | *SLC26A4* | 107330644 | NM_000441.1:c.1225C>T | NP_000432.1:p.(Arg409Cys) |
| PM2+PM3+PP1+PP3 | *SLC26A4* | 107330681 | NM_000441.1:c.1262A>C | NP_000432.1:p.(Gln421Pro) |
| PM2+PM3+PP1+PP3 | *SLC26A4* | 107335067 | NM_000441.1:c.1343C>G | NP_000432.1:p.(Ser448Trp) |
| PVS1+PM2 | *TPRN* | 140094005 | NM_001128228.2:c.1159G>T | NP_001121700.2:p.(Glu387*) |
| PVS1+PM2 | *TRIOBP* | 38106450 | NM_001039141.2:c.131C>G | NP_001034230.1:p.(Ser44*) |
| PVS1+PM2 | *TRIOBP* | 38119905 | NM_001039141.2:c.826C>T | NP_001034230.1:p.(Arg276*) |
| PVS1+PM2 | *USH1C* | 17522624 | NM_005709.3:c.1452_1453delGT | [NP_005700.2](https://www.ncbi.nlm.nih.gov/projects/sviewer/?id=NP_005700.2&search=NP_005700.2:p.Glu519Asp&v=1:100&content=5):p.(Tyr485*) |
| PVS1+PM2 | *STRC* | 43893731 | NM_153700.2:c.4564G>T | NP_714544.1:p.(Gly1522*) |
| PVS1+PM2 | *MYO1A* | 57437760 | NM_005379.3:c.772G>T | NP_005370.1:p.(Glu258*) |

**Table S3 Genotypes of sporadic deafness cases with positive SNVs diagnosis**

| Genes | Genotype | | Patients  number |
| --- | --- | --- | --- |
| *GJB2* | NM_004004.5:c.[235delC];[235delC] | NP_003995.2:p.[Leu79Cysfs*3];[Leu79Cysfs*3] | *54* |
|  | NM_004004.5:c.[299del AT];[299del AT] | NP_003995.2:p.[His100Argfs*14];[His100Argfs*14] | *4* |
|  | NM_004004.5:c.[176del16bp];[176del16bp] | NP_003995.2:p.[Gly59Alafs*18];[Gly59Alafs*18] | *1* |
|  | NM_004004.5:c.[235delC];[299delAT] | NP_003995.2:p.[Leu79Cysfs*3];[His100Argfs*14] | *18* |
|  | NM_004004.5:c.[176del16bp];[235delC] | NP_003995.2:p.[Gly59Alafs*18];[Leu79Cysfs*3] | *5* |
|  | NM_004004.5:c.[235delC];[508_511insAACG] | NP_003995.2:p.[Leu79Cysfs*3];[Ala171Glufs*40] | *3* |
|  | NM_004004.5:c.[235delC];[257C>G] | NP_003995.2:p.[Leu79Cysfs*3];[Thr86Arg] | *2* |
|  | NM_004004.5:c.[257C>G];[299delAT] | NP_003995.2:p.[Thr86Arg];[His100Argfs*14] | *1* |
|  | NM_004004.5:c.[35insG];[439G>A] | NP_003995.2:p.[Val13Cysfs*35];[Glu147Lys] | *1* |
|  | NM_004004.5:c.[235delC];[427C>T] | NP_003995.2:p.[Leu79Cysfs*3];[Arg143Trp] | *1* |
|  | NM_004004.5:c.[235delC];[-23+1G>A] | NP_003995.2:p.[Leu79Cysfs*3];splicing site | *1* |
|  | NM_004004.5:c.[235delC];[308_310delAGA] | NP_003995.2:p.[Leu79Cysfs*3];[Lys103del] | *1* |
|  | NM_004004.5:c.[35insG];[235delC] | NP_003995.2:p.[Val13Cysfs*35];[Leu79Cysfs*3] | *1* |
|  | NM_004004.5:c.[35delG];[235delC] | NP_003995.2:p.[Gly12Valfs*2];[Leu79Cysfs*3] | *1* |
|  | NM_004004.5:c.[235delC];[605ins46] | NP_003995.2:p.[Leu79Cysfs*3];[Cys202*] | *1* |
|  | NM_004004.5:c.[109G>A];[235delC] | NP_003995.2:p.[Val37Ile];[Leu79Cysfs*3] | *4* |
|  | NM_004004.5:c.[109G>A];[427C>T] | NP_003995.2:p.[Leu79Cysfs*3];[Arg143Trp] | *1* |
| *SLC26A4* | NM_000441.1: c.[919-2A>G];[919-2A>G] | NP_000432.1:splicing site/splicing site | *21* |
|  | NM_000441.1:c.[2168A>G];[2168A>G] | NP_000432.1:p.[His723Arg];[His723Arg] | *1* |
|  | NM_000441.1:c.[1229C>T];[1229C>T] | NP_000432.1:p.[Thr410Met];[Thr410Met] | *2* |
|  | NM_000441.1:c.[1174A>T];[1174A>T] | NP_000432.1:p.[Asn392Tyr];[Asn392Tyr] | *1* |
|  | NM_000441.1:c.[1174A>T];[1229C>T] | NP_000432.1:p.[Asn392Tyr];[Thr410Met] | *1* |
|  | NM_000441.1:c.[1174A>T];[1262A>C] | NP_000432.1:p.[Asn392Tyr];[Gln421Pro] | *1* |
|  | NM_000441.1:c.[1174A>T];[2168A>G] | NP_000432.1:p.[Asn392Tyr];[His723Arg] | *1* |
|  | NM_000441.1:c.[1174A>T];[2027T>A] | NP_000432.1:p.[Asn392Tyr];[Leu676Gln] | *1* |
|  | NM_000441.1:c.[1225C>T];[1226G>A] | NP_000432.1:p.[Arg409Cys];[Arg409His] | *1* |
|  | NM_000441.1:c.[1226G>A];[2168A>G] | NP_000432.1:p.[Arg409His];[His723Arg] | *2* |
|  | NM_000441.1:c.[1219C>T];[2162C>T] | NP_000432.1:p.[Thr721Met];[Thr721Met] | *1* |
|  | NM_000441.1:c.[919-2A>G];[915dupG] | NP_000432.1:splicing site;p.[Val306Glyfs*24] | *1* |
|  | NM_000441.1:c.[919-2A>G];[907G>C] | NP_000432.1:splicing site;p.[Glu303Gln] | *1* |
|  | NM_000441.1:c.[919-2A>G];[2168A>G] | NP_000432.1:splicing site;p.[His723Arg] | *12* |
|  | NM_000441.1:c.[919-2A>G];[1174A>T] | NP_000432.1:splicing site;p.[Asn392Tyr] | *2* |
|  | NM_000441.1:c.[919-2A>G];[1225C>T] | NP_000432.1:splicing site;p.[Arg409Cys] | *1* |
|  | NM_000441.1:c.[919-2A>G];[1226G>A] | NP_000432.1:splicing site;p.[Arg409His] | *3* |
|  | NM_000441.1:c.[919-2A>G];[1229C>T] | NP_000432.1:splicing site;p.[Thr410Met] | *1* |
|  | NM_000441.1:c.[919-2A>G];[1219C>T] | NP_000432.1:splicing site;p.[Leu407Phe] | *1* |
|  | NM_000441.1:c.[919-2A>G];[918+2T>C] | NP_000432.1:splicing site/splicing site | *1* |
|  | NM_000441.1:c.[919-2A>G];[1975G>C] | NP_000432.1:splicing site;p.[Val659Leu] | *3* |
|  | NM_000441.1:c.[919-2A>G];[2086C>T] | NP_000432.1:splicing site;p.[Gln696*] | *1* |
|  | NM_000441.1:c.[919-2A>G];[1340delA] | NP_000432.1:splicing site;p.[Lys447Serfs*8] | *2* |
|  | NM_000441.1:c.[919-2A>G];[1544+9C>T] | NP_000432.1:splicing site/splicing site | *1* |
|  | NM_000441.1:c.[919-2A>G];[109G>T] | NP_000432.1:splicing site;p.[Glu37*] | *1* |
|  | NM_000441.1:c.[919-2A>G];[404A>G] | NP_000432.1:splicing site;p.[His135Arg] | *1* |
|  | NM_000441.1:c.[919-2A>G];[1614+1G>A] | NP_000432.1:splicing site/splicing site | *1* |
|  | NM_000441.1:c.[919-2A>G];[281C>T] | NP_000432.1:splicing site;p.[Thr94Ile] | *1* |
|  | NM_000441.1:c.[919-2A>G];[304G>A] | NP_000432.1:splicing site;p.[Gly102Arg] | *1* |
|  | NM_000441.1:c.[919-2A>G];[235C>T] | NP_000432.1:splicing site;p.[Arg79*] | *1* |
|  | NM_000441.1:c.[919-2A>G];[589G>A] | NP_000432.1:splicing site;p.[Gly197*] | *1* |
|  | NM_000441.1:c.[919-2A>G];[754T>C] | NP_000432.1:splicing site;p.[Ser252Pro] | *1* |
|  | NM_000441.1:c.[919-2A>G];[563T>C] | NP_000432.1:splicing site;p.[Ile188Thr] | *1* |
|  | NM_000441.1:c.[1975G>C];[2168A>G] | NP_000432.1:p.[ Val659Leu] ;[His723Arg] | *2* |
|  | NM_000441.1:c.[1707+5G>A];[1318A>T] | NP_000432.1:splicing site;p.[Lys440*] | *1* |
|  | NM_000441.1:c.[1673A>T];[1686insA] | NP_000432.1:p.[Asn558Ile];[Cys565Metfs*9] | *1* |
|  | NM_000441.1:c.[281C>T];[2168A>G] | NP_000432.1:p.[Thr94Ile];[His723Arg] | *1* |
|  | NM_000441.1:c.[589G>A];[1229C>T] | NP_000432.1:p[.Gly197Arg];[Gly197Arg] | *1* |
|  | NM_000441.1:c.[563T>C];[1746delG] | NP_000432.1:p.[Ile188Thr];[Ala584Argfs*2] | *1* |
|  | NM_000441.1:c.[754T>C];[1720G>A] | NP_000432.1:p.[Ser252Pro];[Ala574Thr] | *1* |
|  | NM_000441.1:c.[919-2A>G];[1079C>T] | NP_000432.1:splicing site;p.[Ala360Val] | *1* |
|  | NM_000441.1:c.[334C>T];[1343C>G] | NP_000432.1:p.[Pro112Ser];[Ser448Trp] | *1* |
|  | NM_000441.1:c.[1174A>T];[1520delT] | NP_000432.1:p.[Asn392Tyr];[Leu507*] | *1* |
|  | NM_000441.1:c.[812A>G];[1466C>T] | NP_000432.1:p.[Asp271Gly];[Ser489Phe] | *1* |
| *12SrRNA* | m.1555A>G homoplasmy |  | *4* |
|  | m.1494C>T homoplasmy |  | *1* |
| *POU3F4* | NM_000307.4:c.[170G>A];[170G>A] | NP_000298.3:p.[Trp57*];[Trp57*] | *1* |
|  | NM_000307.4:c.[985C>G];[985C>G] | NP_000298.3:p.[Arg329Gly];[Arg329Gly] | *1* |
|  | NM_000307.4:c.[700A>T];[700A>T] | NP_000298.3:p.[Arg234Trp];[Arg234Trp] | *1* |
| *KCNQ1* | NM_000218.2:c.[533G>A];[533G>A] | NP_000209.2:p.Trp178*];[Trp178*] | *1* |
| *KCNQ4* | NM_004700.3:c.[573G>A] | NP_004691.2:p.[Gly192Asp] | *1* |
|  | NM_004700.3:c.[1428T>C] | NP_004691.2:p.[Met477Thr] | *1* |
| *PAX3* | NM_181457.3:c.[811C>T] | [NP_852123.1](https://www.ncbi.nlm.nih.gov/projects/sviewer/?id=NP_852123.1&search=NP_852123.1:p.Gly43&v=1:100&content=5):p.[Arg271Cys] | *1* |
| *MITF* | NG_011631.1(NM_000248.3):c.[710+1G>A] | splicing site | *1* |
| *MYO6* | NM_004999.3:c.[2840G>A] | NP_004990.3:p.[Arg947Gln] | *1* |
| *OTOF* | NM_194248.2:c.[4994T>C];[2797G>C] | NP_919224.1:p.[Leu1665Pro];[Glu933Gln] | *1* |
| *TECTA* | NM_005422.2 :c.[2332G>A];[5488G>A] | NP_005413.2 :p.[Val778Ile];[Val1830Met] | *1* |
| *PDZD7* | NM_001195263.1:c.[2368_2377delAAGTCTCCAG];  [1207delC] | NP_001182192.1:p.[Lys790Valfs*15];[His403Ilefs*36] | *1* |
| *CLDN14* | NM_001146077.1:c.[314C>T];[694G>A] | NP_001139549.1:p.[Thr105Met];[Gly232Arg] | *1* |
| *OTOA* | NM_144672.4:c.[35T>G];[1736T>G] | NP_653273.3:p.[Leu12Arg];[Met579Arg] | *1* |
| *CDH23* | NM_022124.5:c.[7145G>A];[9082A>T] | NP_071407.4:p.[Arg2382Gln];[Ile3028Phe] | *1* |
|  | NM_022124.5:c.[995C>A];[6049G>A] | NP_071407.4:p.[Thr332Lys];[Gly2017Ser] | *1* |
| *MYO15A* | NM_016239.3:c.[8791delT];[6611G>A] | NP_057323.3:p.[Trp2931Glyfs*103];[Arg2204His] | *1* |
|  | NM_016239.3:c.[5023T>C];[8091+1G>A] | NP_057323.3:p.[Phe1675Leu];splicing site | *1* |
|  | NM_016239.3:c.[8033_8046delATCCCAACTTCTAC];  [9690+1G>A] | NP_057323.3:p.[Asn2678Argfs*29];splicing site | *1* |
|  | NM_016239.3:c.[9572G>A];[6610C>T] | NP_057323.3:p.[Arg3191His];[Arg2204Cys] | *1* |
| *TMC1* | NM_138691.2:c.[624C>A];[1189G>A] | NP_619636.2:p.[Ser208Arg];[Asp397Asn] | *1* |
|  | NM_138691.2:c.[589G>A];[236+1G>A] | NP_619636.2:p.[Gly197Arg]/splicing site | *1* |
|  | NM_138691.2:c.[1298A>C];[100C>T] | NP_619636.2:p.[Tyr433Ser];[Arg34*] | *1* |
| *ADGRV1* | NM_032119.3:c.[15505A>T];[3509A>G] | NP_115495.3:p.[Ile5169Phe];[Tyr1170Cys] | *1* |
| *TRIOBP* | NM_001039141.2:c.[5045A>G];[6067G>T] | NP_001034230.1:p.[Tyr1682Cys];[Val2023Leu] | *1* |
|  | NM_001039141.2:c.[1075G>A];[5248C>T] | NP_001034230.1:p[.Ala359Thr];[Arg1750Trp] | *1* |
| *MYO1F* | NM_012335.3 :c.[2201G>A];[373G>T] | NP_036467.2 :p.[Arg734Gln];[Gly125Cys] | *1* |
|  | NM_012335.3 :c.[2276G>A];[941T>C] | NP_036467.2 :p.[Arg759Gln];[Ile314Thr] | *1* |
|  | NM_012335.3 :c.[3163G>A];[2129T>A] | NP_036467.2 :p.[Val1055Met];[Val710Glu] | *1* |
| *USH2A* | NM_206933.2:c.[14017T>C];[2802T>G] | NP_996816.2:p.[Tyr4673His];[Cys934Trp] | *1* |
|  | NM_206933.2:c.[12388delC];[436delG] | NP_996816.2:p.[Leu4130*];[Ala146Hisfs*5] | *1* |
|  | NM_206933.2:c.[6998T>C];[2855C>T] | NP_996816.2:p.[Val2333Ala];[Ser952Leu] | *1* |
| *MYO7A* | NM_000260.3:c.[1258A>T];[1258A>T] | NP_000251.3:p.[Lys420*];[Lys420*] | *1* |
|  | NM_000260.3:c.[3576G>A];[390_391insC] | NP_000251.3:p.[Trp1192*];[His133Profs*7] | *1* |
|  | NM_000260.3:c.[5287G>A];[5733_5735delCTT] | NP_000251.3:p.[Glu1763Lys];[Phe1914del] | *1* |
| *TRPN* | NM_001128228.2:c.[1050C>G];[862G>A] | NP_001121700.2:p.[Ile350Met];[Val288Met] | *1* |
| *TMPRSS3* | [NM_024022.2](https://www.ncbi.nlm.nih.gov/clinvar/variation/46104/):c.[1558C>T];[1300+1G>A] | NP_076927.1:p.[Arg520Cys;/splicing site | *1* |
|  |  |  |  |

**Table S4 Allele Frequency and Carrier Frequency for Common Deafness Gene Variants**

| **Gene** | **Inheritance** | **Common variants** | **Allele frequency**  **in patients** | **Allele frequency**  **in controls** | **Carrier frequency** | **MAF in ExAc databases** |
| --- | --- | --- | --- | --- | --- | --- |
| *GJB2* | AR | **c.235delC p.(Leu79Cysfs)** | **18.13%(157/864)** | **0.49%(6/1232)** | **0.97%(6/616)** | **NA** |
|  |  | **c.109G>A p.(Val37Ile)** | **3.81% (33/864)** | **3.00%(37/1232)** | **5.84%(36/616)** | **0.0013** |
|  |  | **c.299_300delAT p.(His100Argfs)** | **3.46%(30/864)** | **0.08%(1/1232)** | **0.16%(1/616)** | **NA** |
|  |  | **c.176_191del16bp**  **p.(Gly59Alafs*18)** | **0.69%(6/864)** | **0.08%(1/1232)** | **0.16%(1/616)** | **NA** |
|  |  | **c.508_511dupAACG**  **p.(Ala171Glufs*40)** | **0.46%(4/864)** | **0(0/1232)** | **0(0/616)** | **NA** |
|  |  | **c.257C>G p.(Thr86Arg)** | **0.35%(3/864)** | **0(0/1232)** | **0(0/616)** | **NA** |
|  |  | c.35delG p.(Gly12Valfs*2) | 0.23%(2/864) | 0(0/1232) | 0(0/616) | 0.0074 |
|  |  | c.35dupG p.(Val13Cysfs*35) | 0.23%(2/864) | 0(0/1232) | 0(0/616) | 0.0074 |
|  |  | c.427C>T p.(Arg143Trp) | 0.23%(2/864) | 0(0/1232) | 0(0/616) | 0.0002 |
|  |  | c.-23+1G>A | 0.12%(1/864) | 0(0/1232) | 0(0/616) | NA |
|  |  | c.9G>A p.(Trp3*) | 0.12% (1/864) | 0(0/1232) | 0(0/616) | NA |
|  |  | c.308_310delAGA p.(Lys103del) | 0.12% (1/864) | 0(0/1232) | 0(0/616) | NA |
|  |  | c.439G>A p.(Glu147Lys) | 0.12% (1/864) | 0(0/1232) | 0(0/616) | NA |
| *SLC26A4* | AR | **c.919-2A>G** | **10.28%(89/864)** | **0.89%(11/1232)** | **1.79%(11/616)** | **NA** |
|  |  | **c.2168A>G p.(His723Arg)** | **2.19%(19/864)** | **0.24%(3/1232)** | **0.49%(3/616)** | **NA** |
|  |  | **c.1174A>T p.(Asn392Tyr)** | **1.27%(11/864)** | **0(0/1232)** | **0(0/616)** | **NA** |
|  |  | **c.1229C>T p.(Thr410Met)** | **0.92%(8/864)** | **0.08%(1/1232)** | **0.16%(1/616)** | **0.0002** |
|  |  | **c.1226G>A p.(Arg409His)** | **0.69%(6/864)** | **0(0/1232)** | **0(0/616)** | **NA** |
|  |  | **c.1975G>C p.(Val659Leu)** | **0.69%(6/864)** | **0.16%(2/1232)** | **0.32%(2/616)** | **NA** |
|  |  | c.1720G>A p.(Ala574Thr) | 0.23%(2/864) | 0.08%(1/1232) | 0.16%(1/616) | NA |
|  |  | c.2027T>A p.(Leu676Gln) | 0.23%(2/864) | 0.08%(1/1232) | 0.16%(1/616) | NA |
|  |  | c.1225C>T p.(Arg409Cys) | 0.23%(2/864) | 0(0/1232) | 0(0/616) | NA |
|  |  | c.754T>C p.(Ser252Pro) | 0.23%(2/864) | 0(0/1232) | 0(0/616) | NA |
|  |  | c.281C>T p.(Thr94Ile) | 0.23%(2/864) | 0(0/1232) | 0(0/616) | NA |
|  |  | c.1340delA p.(Lys447Serfs*8) | 0.23%(2/864) | 0(0/1232) | 0(0/616) | NA |
|  |  | c.1707+5G>A | 0.12%(1/864) | 0(0/1232) | 0(0/616) | 0.000249 |
|  |  | c.1318A>T p.(Lys440*) | 0.12%(1/864) | 0(0/1232) | 0(0/616) | NA |
|  |  | c.589G>T p.(Gly197*) | 0.12%(1/864) | 0(0/1232) | 0(0/616) | NA |
|  |  | c.109G>T p.(Glu37*) | 0.12%(1/864) | 0(0/1232) | 0(0/616) | NA |
|  |  | c.918+2T>C | 0.12%(1/864) | 0(0/1232) | 0(0/616) | NA |
|  |  | c.915dupG  p.(Val306Glyfs*24) | 0.12%(1/864) | 0(0/1232) | 0(0/616) | NA |
|  |  | c.1686_1687insA  p.(Cys565Metfs*9) | 0.12%(1/864) | 0(0/1232) | 0(0/616) | NA |
|  |  | c.1519delT p.(Leu507*) | 0.12%(1/864) | 0(0/1232) | 0(0/616) | NA |
|  |  | c.235C>T p.(Arg79*) | 0.12%(1/864) | 0(0/1232) | 0(0/616) | NA |
|  |  | c.2086C>T p.(Gln696*) | 0.12%(1/864) | 0(0/1232) | 0(0/616) | NA |
|  |  | c.2162C>T p.(Thr721Met) | 0.12%(1/864) | 0(0/1232) | 0(0/616) | NA |
|  |  | c.1079C>T p.(Ala360Val) | 0.12%(1/864) | 0(0/1232) | 0(0/616) | NA |
|  |  | c.85G>C p.(Glu29Gln) | 0.12%(1/864) | 0(0/1232) | 0(0/616) | NA |
|  |  | c.1746delG  p.(Ala584Argfs*2) | 0.12%(1/864) | 0(0/1232) | 0(0/616) | NA |
|  |  | c.697G>C p.(Val233Leu) | 0.12%(1/864) | 0(0/1232) | 0(0/616) | NA |
| *KCNQ1* | AR | c.533G>A p.(Trp178*) | 0.23%(2/864) | 0(0/1232) | 0(0/616) | NA |
| ***POU3F4*** | **XLR** | **c.170G>A p.(Trp57*)** | **0.23% (2/864)** | **0(0/1232)** | **0(0/616)** | **NA** |
|  |  | **c.985C>G p.(Arg329Gly)** | **0.23% (2/864)** | **0(0/1232)** | **0(0/616)** | **NA** |
|  |  | **c.700A>T p.(Arg234Trp)** | **0.23% (2/864)** | **00/1232)** | **0(0/616)** | **NA** |
| *EDNRB* | AR | c.1247G>A p.(Trp416*) | 0.23% (2/864) | 0(0/1232) | 0(0/616) | NA |
| *CDH23* | AR | c.6604G>A p.(Asp2202Asn) | 0.23% (2/864) | 0(0/1232) | 0(0/616) | NA |
| ***MYO7A*** | **AR** | **c.4439C>A p.(Ser1480*)** | **0.35%(3/864)** | **0(0/1232)** | **0(0/616)** | **NA** |
|  |  | c.1258A>T p.(Lys420*) | 0.23% (2/864) | 0(0/1232) | 0(0/616) | NA |
| ***TMC1*** | **AR** | **c.100C>T p.(Arg34*)** | **0.35% (3/864)** | **0(0/1232)** | **0(0/616)** | **NA** |
|  |  | c.2218G>T p.(Glu740*) | 0.12% (1/864) | 0.08%(1/1232) | 0(0/616) | NA |
| ***OTOA*** | **AR** | **c.1387G>T p.(Glu463*)** | **0.69%(6/864)** | **0.16%(2/1232)** | **0.32%(2/616)** | **NA** |
| ***USH2A*** | **AR** | **c.6998T>C p.(Val2333Ala)** | **1.04%(9/864)** | **0(0/1232)** | **0(0/616)** | **0.0001** |
|  |  | **c.2802T>G p.(Cys934Trp)** | **0.69% (6/864)** | **0.24%(3/1232)** | **0.49%(3/616)** | **NA** |
| *CATSPER2* | AR | c.1574T>A p.(Leu525*) | 0.23%(2/864) | 0(0/1232) | 0(0/616) | NA |
|  | | | | | | |

The overstriking variants were the top 20 variants that affect protein function.
